# Primary and secondary transcriptional effects in the developing human Down syndrome brain and heart

**DOI:** 10.1186/gb-2005-6-13-r107

**Published:** 2005-12-16

**Authors:** Rong Mao, Xiaowen Wang, Edward L Spitznagel, Laurence P Frelin, Jason C Ting, Huashi Ding, Jung-whan Kim, Ingo Ruczinski, Thomas J Downey, Jonathan Pevsner

**Affiliations:** 1 Program in Biochemistry, Cellular and Molecular Biology, Johns Hopkins School of Medicine, 1830 East Monument Street, Baltimore, MD 21205, USA; 2 Department of Neuroscience, Johns Hopkins School of Medicine, 725 North Wolfe Street, Baltimore, MD 21205, USA; 3 Partek Incorporated, St Charles, MO 63304, USA; 4 Department of Mathematics, Campus Box 1146, Washington University, St Louis, MO 63130, USA; 5 Department of Neurology, Kennedy Krieger Institute, 707 North Broadway, Baltimore, MD 21205, USA; 6 Pathobiology Graduate Program, Johns Hopkins School of Medicine, 720 Rutland Avenue, Baltimore, MD 21205, USA; 7 Department of Biostatistics, Johns Hopkins Bloomberg School of Public Health, 615 North Wolfe Street, Baltimore, MD 21205, USA

## Abstract

**Background:**

Down syndrome, caused by trisomic chromosome 21, is the leading genetic cause of mental retardation. Recent studies demonstrated that dosage-dependent increases in chromosome 21 gene expression occur in trisomy 21. However, it is unclear whether the entire transcriptome is disrupted, or whether there is a more restricted increase in the expression of those genes assigned to chromosome 21. Also, the statistical significance of differentially expressed genes in human Down syndrome tissues has not been reported.

**Results:**

We measured levels of transcripts in human fetal cerebellum and heart tissues using DNA microarrays and demonstrated a dosage-dependent increase in transcription across different tissue/cell types as a result of trisomy 21. Moreover, by having a larger sample size, combining the data from four different tissue and cell types, and using an ANOVA approach, we identified individual genes with significantly altered expression in trisomy 21, some of which showed this dysregulation in a tissue-specific manner. We validated our microarray data by over 5,600 quantitative real-time PCRs on 28 genes assigned to chromosome 21 and other chromosomes. Gene expression values from chromosome 21, but not from other chromosomes, accurately classified trisomy 21 from euploid samples. Our data also indicated functional groups that might be perturbed in trisomy 21.

**Conclusions:**

In Down syndrome, there is a primary transcriptional effect of disruption of chromosome 21 gene expression, without a pervasive secondary effect on the remaining transcriptome. The identification of dysregulated genes and pathways suggests molecular changes that may underlie the Down syndrome phenotypes.

## Abstract 2

Microarray analysis of transcript levels in fetal cerebellum and heart tissues of Down syndrome patients showed a disruption only of chromosome 21 gene expression.

## Background

Human autosomal abnormality is the leading cause of early pregnancy loss, neonatal death, and multiple congenital malformations [1,2]. Among all the autosomal aneuploidies, Down syndrome (DS), with an incidence of 1 in approximately 800 live births, is most frequently compatible with postnatal survival. It is characterized by mental retardation, hypotonia, short stature, and several dozen other anomalies [3-5].

It has been known since 1959 that DS is caused by the triplication of a G group chromosome, now known to be human chromosome 21 [6,7]. As for all aneuploidies, the phenotype of DS is thought to result from the dosage imbalance of multiple genes. By the 1980s, a primary effect of increased gene products, proportional to gene dosage, was established for dozens of enzymes in studies of various aneuploidies [5]. More recently, microarrays and other high-throughput technologies have allowed the measurement of steady-state RNA levels for thousands of transcripts in human DS cells [8-10] and in tissues obtained from mouse models of DS [11-15]. Most of these studies have confirmed a primary gene dosage effect. We previously measured RNA transcript levels in fetal trisomic and euploid cerebrum samples, and in astrocyte cell lines derived from cerebrum [16]. We observed a dramatic, statistically significant increase in the expression of trisomic genes assigned to chromosome 21.

The secondary, downstream consequences of aneuploidy are complex. A major unanswered question is the extent to which secondary changes occur in DS as a consequence of the aneuploid state. On chromosome 21, gene expression may be regulated by dosage compensation or other mechanisms such that only a subset of those genes is expressed at the expected 50% increased levels. For genes assigned to chromosomes other than 21, the effect of trisomy 21 (TS21) could be relatively subtle or massively disruptive. It has been hypothesized that gene expression changes in chromosome 21 are likely to affect the expression of genes on other chromosomes through the modulation of transcription factors, chromatin remodeling proteins, or related molecules [5,17,18]. Recent studies in human and in mouse provide conflicting evidence, with some studies suggesting only limited effects of trisomy on the expression of disomic genes, whereas other studies indicate pervasive effects (see Discussion).

In the present study, we assessed five specific hypotheses relating to primary and secondary transcriptional changes in DS. First, which, if any, chromosomes exhibited overall differential expression between TS21 and controls? Our previous study in human tissue [8,16] suggested the occurrence of dosage-dependent transcription for chromosome 21 genes, but not for genes assigned to other chromosomes. The present report addressed whether this phenomenon applies to multiple tissues in DS.

Second, which, if any, genes assigned to chromosome 21 exhibited differential expression between TS21 and controls? Third, which, if any, genes on chromosomes other than chromosome 21 exhibited differential expression between TS21 and controls? Previous studies by other groups [8,9,19,20] and by us [16] lacked sufficient statistical power to identify significantly regulated genes in DS. The present study identified such genes by using a larger sample size, by combining previous data from cerebrum and astrocytes [16] with gene expression data from additional tissue types (cerebellum and heart), and by using analysis of variance (ANOVA).

Fourth, can we classify tissue samples as TS21 or controls using genes on chromosome 21 or genes on chromosomes other than 21? Classification is a supervised learning technique that provides a powerful statistical approach to address the question whether only chromosome 21 or the entire transcriptome is involved in DS. Fifth, which, if any, functional groups of genes exhibited overall differential expression between TS21 and controls? Such analysis may reveal biological processes that are perturbed in DS.

In this study we measured gene expression in heart and cerebellum, two regions that are pathologically affected in DS. Total brain volume is consistently reduced in DS, with a disproportionately greater reduction in the cerebellum [21,22]. Furthermore, a significant reduction in granule cell density in the DS cerebellum has been reported for both human and the Ts65Dn mouse model of DS [23]. Another prominent phenotype of DS is congenital heart defects. TS21 has the highest association with major heart abnormalities among all chromosomal defects, and 40% to 50% of TS21 children have heart defects [24,25]. Of those children with heart abnormalities, 44% to 48% are specifically affected with atrial ventricular septal defects (AVSDs) [26]. Other commonly affected tissues in the DS heart include the valve regions, such as pulmonary and mitral valves [27,28]. Barlow *et al*. [29] assessed congenital heart disease in DS patients with partial duplications of chromosome 21, and established a critical region of over 50 genes. The expression levels of these genes in fetal TS21 heart samples have not yet been assessed.

Our data showed consistent, statistically significant overall dosage-dependent expression of genes assigned to chromosome 21. Analysis of these data identified genes with most consistent dysregulation of expression in different TS21 fetal tissue and cell types, most of which were independently confirmed by quantitative real-time PCR. We successfully classified tissue samples using expression data from chromosome 21 genes, but not with the data on non-chromosome 21 genes. Statistical analyses on our microarray data also indicated tissue-specific, regulated functional groups of genes, which may provide initial clues to perturbed biological pathways in TS21. Overall, the data support a model in which the aneuploid state increases the expression of chromosome 21 genes, with complex but limited secondary effects on transcript levels of genes on other chromosomes.

## Results

### Exploratory analyses of gene expression

**Figure 1:**
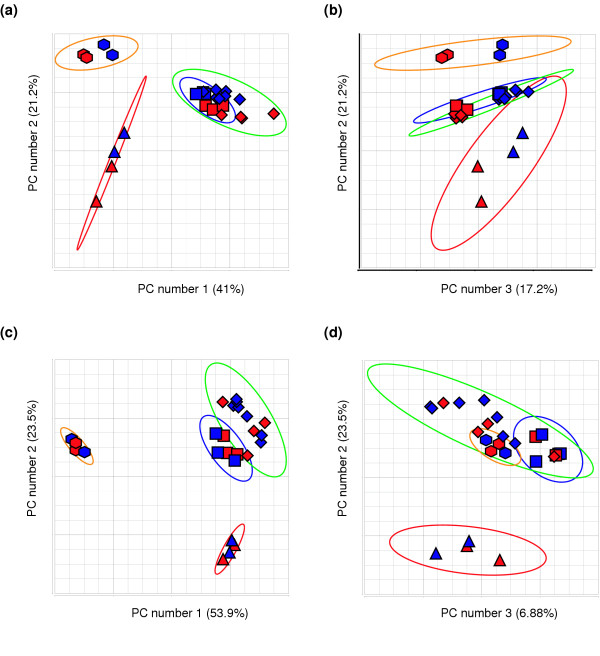
PCA was used to visually assess the major sources of variation in the expression data. For each of the four panels, each data point represents a sample; there are 25 samples total. (**a**) PCA applied to chromosome 21 genes. The x-axis represents the first PC (accounting for 41% of the variance) and the y-axis represents the second PC (accounting for 21.2%). The graph is based on expression values for all 253 probe sets assigned to chromosome 21. This showed that the largest source of variability was due to tissue/cell type, accounting for 62.2% of the variance in the data. (**b**) PCA applied to chromosome 21 genes. The x-axis corresponds to the third PC, and the y-axis corresponds to the second PC. The third PC showed a separation of trisomic from euploid samples based on gene expression, accounting for 17.2% of the variance in the data. (**c**) PCA applied to non-chromosome 21 genes. The first two PCs (x- and y-axis) using expression values for genes assigned to all other chromosomes also showed that the largest source of variance was due to tissue (77.4% of total variance). These observations are similar to the results in panel a. (**d**) PCA applied to non-chromosome 21 genes. The x- and y-axis correspond to the third and second PCs, respectively. In contrast to the results of panel b, the third PC failed to show separation of trisomic from euploid samples (6.9% of total variance). The ellipsoids represent three standard deviations beyond the centroid of each tissue group. Data points correspond to samples (red, Down syndrome; blue, euploid) within a group (cerebrum, diamond symbols on data points, and green ellipsoid; cerebellum, square symbols on data points and blue ellipsoid; astrocyte, triangle symbols on data points and red ellipsoid; heart, hexagon symbols on data points and orange ellipsoid).

We measured the expression levels of up to 18,462 transcripts, representing approximately 15,106 genes, using Affymetrix GeneChip^® ^human U133A microarrays. These transcripts corresponded to 20,261 probe sets, excluding 2,023 Affymetrix bacterial and housekeeping control probes and probes that do not map to any chromosomes. We performed principal components analysis (PCA) to explore the gene expression profiles from four regions (cerebrum, cerebellum, heart, and cerebrum-derived astrocyte cell lines) in human fetal samples diagnosed with TS21 and matched euploid controls (see Additional data file 1). PCA allows the visualization of highly dimensional data along principal component (PC) axes. These axes reflect the degree of variance in the data, allowing the identification of groups of data points having possible biological relevance. For example, two points corresponding to tissue samples that are close together in PCA space are likely to have highly similar overall gene expression profiles. Figure 1 shows the 25 tissue samples mapped from high-dimensional space to three dimensions for exploratory visualization. The first three PCs are displayed on the x-, y-, and z-axes, respectively. The percentage of total variance explained by each PC is displayed on the corresponding axis. This analysis was performed on 253 probe sets (chromosome 21) and 20,008 probe sets (non-chromosome 21) separately. Figure 1 shows that for chromosome 21 and non-chromosome 21 genes, the samples clustered primarily by tissue or cell type. Thus, the largest differences in overall gene expression between the samples exhibited by PCA are attributable to the different tissues or cells. For genes on chromosome 21, TS21 is distinguishable from euploid controls on the third PC, which accounts for 17.2% of the total variation in 253-dimensional data (Figure 1b). In contrast, PCA mapping of non-chromosome 21 genes (Figure 1c,d) showed no distinction between TS21 and euploid controls. Although only the first three PCs are displayed in Figure 1, a difference between TS21 and euploid controls was not significant on any of the PCs (based on a *t* test performed on each PC; data not shown).

**Figure 2:**
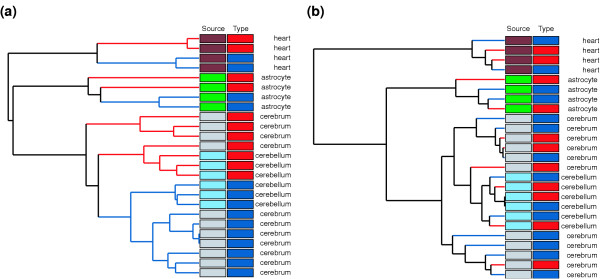
Dendrograms from hierarchical clustering. Dendrograms were based on (**a**) chromosome 21 genes and (**b**) non-chromosome 21 genes in the 25 samples, using Euclidean distance and average linkage. Branch lengths represent dissimilarity. Samples were of two types (TS21, red; euploid, dark blue) and four sources (astrocyte, green; cerebellum, light blue; cerebrum, gray; heart, brown).

To further explore the relationships between samples based upon gene expression profiles, we performed hierarchical clustering using average linkage with Euclidean distance (Figure 2). Hierarchical clustering and PCA are 'unsupervised' methods, which do not consider the known sample attributes such as tissue type or disease state when organizing the data. We superimposed the sample information using color coding. Consistent with PCA, cluster analysis indicated that the samples clustered primarily by tissue source in both chromosome 21 genes and non-chromosome 21 genes. The clustering for the chromosome 21 genes showed a tendency to cluster by disease type within the tissue clusters (Figure 2a), whereas no obvious clustering by disease type was evident in the primary clusters or sub-clusters of genes not on chromosome 21 (Figure 2b). Cluster analysis and PCA results are consistent with the hypothesis that TS21 samples are distinguishable from matched euploid samples based upon differences in the expression of genes assigned to chromosome 21. Additionally, these exploratory analyses revealed no substantial outliers or other anomalies in the data.

### Statistical testing of gene expression

We used a mixed-model ANOVA to test the first three hypotheses stated in the introduction. The hypotheses tested included multiple tests on chromosomes or individual genes. Therefore, to protect against false discoveries due to multiple testing, we used the step-up 'false discovery rate' (FDR) [30]. We set the FDR at 0.05, meaning that the list of significant genes after applying FDR is expected to contain 5% false positives.

**Figure 3:**
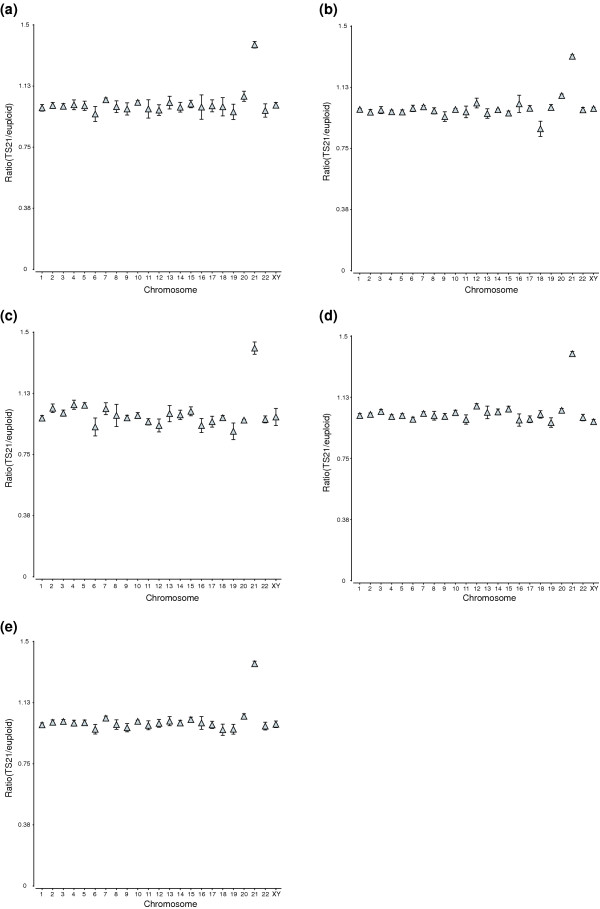
Increased transcript levels of genes assigned to chromosome 21 in TS21 samples compared to controls. The plots show ratio (TS21/euploid) of mean expression values, calculated using data from samples in each tissue or cell type, for all 23 chromosomes. (X and Y chromosome data were pooled.) The expression values were obtained with Affymetrix MAS5 software. The error bars represent standard errors (obtained by performing 1,000 iterations of a bootstrap resampling of the tissues). (**a**) The ratio of TS21 to euploid mean expression values for each chromosome in fetal cerebrum samples. (**b**) The ratio of TS21 to euploid mean expression values in fetal cerebellum samples. (**c**) The ratio of TS21 to euploid mean expression values in cultured astrocyte cell lines derived from fetal cerebrum tissues. (**d**) The ratio of TS21 to euploid mean expression values in fetal heart samples. (**e**) The ratio of TS21 to euploid mean expression values using data from all the above tissue and cell types.

For the first hypothesis, we assessed whether genes assigned to each chromosome displayed overall differential gene expression. Only chromosome 21 showed significant mean overall differential expression between TS21 and euploid controls (Figure 3). Genes on chromosome 21 were expressed at 1.37 ± 0.02 fold (mean ± standard error), while the ratio of TS21/control across the other chromosomes was 1.00 ± 0.02 (ranging from 0.96 ± 0.03 to 1.02 ± 0.03). For this first hypothesis, 23 chromosomes were tested (chromosomes X and Y were combined), so the FDR is based on n = 23 tests.

For the second hypothesis, we tested whether individual genes assigned to chromosome 21 were differentially expressed in TS21 versus euploid samples. A mixed-model ANOVA (see Materials and methods) identified 26 out of 253 chromosome 21 probe sets (10.2%) with statistically significant differential expression at a FDR of 0.05. These most consistently dysregulated genes are listed in Table 1. For 104 gene expression comparisons listed in Table 1, 103 were increased in TS21 relative to controls. For this hypothesis, the FDR was based on n = 253 tests (for the number of probe sets assigned to chromosome 21).

For the third hypothesis, we tested whether individual genes not assigned to chromosome 21 were differentially expressed in TS21 relative to euploid samples. The presence of such genes would indicate whether the condition of TS21 causes changes in the transcriptome on chromosomes other than 21, possibly as a secondary consequence of the trisomy. Out of 20,008 non-chromosome 21 probe sets, 14 exhibited statistically significant differential expression at a FDR of 0.05 (Table 2). Using an alternative approach, we performed FDR on each chromosome separately with similar results (Additional data file 2). The same 14 genes passed FDR at the 0.05 level, as well as three additional genes (2,4-dienoyl CoA reductase 1 (NM_001359) and cholinergic receptor, nicotinic, alpha polypeptide 2 (NM_000742), both assigned to chromosome 8, and small inducible cytokine subfamily A (Cys-Cys), member 21 (NM_002989), assigned to chromosome 9). For chromosome 21 genes, 10.3% passed FDR at 0.05; for all other chromosomes, the greatest number of genes passing was 0.3% (chromosome 18) (Additional data file 2).

**Figure 4:**
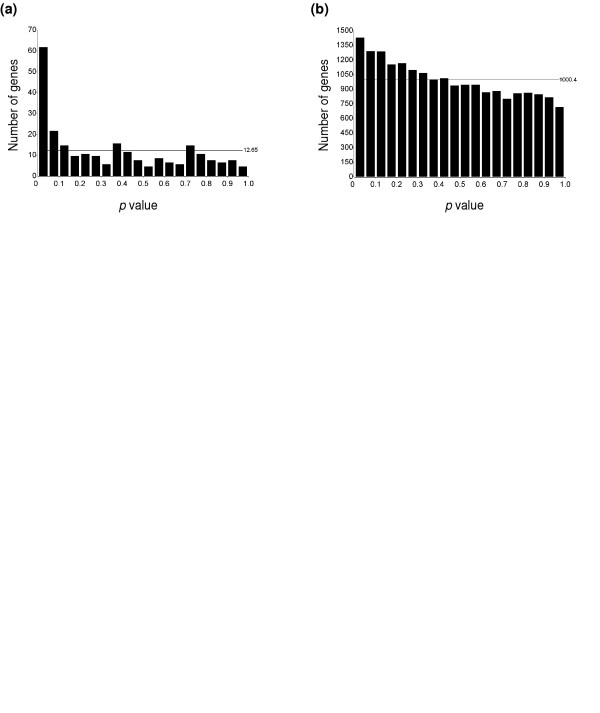
Histograms of *p *values. (**a**) Distribution of *p *values for chromosome 21 genes (253 probe sets represented on the microarray). The histogram contains 20 bins, at intervals of 0.05. The expected number of genes in each bin by chance alone is 253 × 0.05 = 12.65 (horizontal line). (**b**) Distribution of *p *values for non-chromosome 21 genes (20,008 probe sets). The expected number of genes having a *p *value < 0.05 by random chance is 20,008 × 0.05 = 1000.4 (horizontal line).

Based on the mixed-model ANOVA, a large proportion of chromosome 21 genes (n = 26 probe sets/253) showed significant altered expression at a FDR of 0.05, while a very small proportion of non-chromosome 21 genes (n = 14 probe sets/20,008) were significantly regulated. We further visualized this phenomenon by plotting a histogram of all the *p *values obtained for chromosome 21 genes (n = 253; Figure 4a) and for non-chromosome 21 genes (n = 20,008; Figure 4b). The histogram in Figure 4a contains 20 bins, at intervals of 0.05. If there were no truly differentially regulated genes, each bin would contain 253 × 0.05 = 12.65 transcripts (horizontal line on the figure). The figure indicates that there are many more small *p *values than expected by chance; there are 62 transcripts with *p *< 0.05, while only about 13 would be expected to be less than 0.05 by chance. For non-chromosome 21 genes (Figure 4b), the expected number of genes having a *p *value less than 0.05 by chance was 1000.4 (20,008 × 0.05), whereas the observed number of genes having *p *< 0.05 was 1,419. Although there was some tendency for the *p *values to be smaller than expected by chance, these two histograms provide a visual display of the extent to which the expression of many chromosome 21 genes are significantly different between TS21 and controls, whereas few genes assigned to other chromosomes were significantly regulated.

We asked whether there were regional differences among the significantly regulated genes. For those genes assigned to chromosome 21 (Table 1), the mean ratio of TS21/euploid mRNA level was 1.58 ± 0.05 (mean ± standard error) in the fetal brain tissues and astrocyte cell lines derived from the frontal cortex. Similarly, the TS21/euploid expression ratio in fetal heart was 1.60 ± 0.09 (with the exception of *TMEM1*, for which the TS21/euploid ratio was 9.58). These results are consistent for a gene expression dosage effect caused by trisomy. However, for significantly regulated genes that were not assigned to chromosome 21 (Table 2), a large percent were abundantly expressed and significantly different between TS21 and euploid samples only in the heart, but not in the brain. These genes included myomesin 1, myoglobin, calsequestrin 2, cardiac troponin I and T2, and alpha 1 actin.

### Classification of TS21 and euploid samples

To more completely assess differential gene expression, we investigated the ability to classify tissue samples as TS21 or euploid controls using genes on chromosome 21 and genes on chromosomes other than 21. The accuracy estimate for classification using chromosome 21 genes was 99.91% correct, whereas the estimate for classification using non-chromosome 21 genes was only 48.63% correct. Tables 3 and 4 show the classification results for the nested cross-validation using chromosome 21 genes and those using non-chromosome 21 genes (see Materials and methods and Additional data file 3). As expected, we were able to classify the tissue samples with very high accuracy using chromosome 21 genes (Table 3). The classification accuracy when using non-chromosome 21 genes was, however, approximately equal to the accuracy expected by chance (Table 4).

### Functional group analysis

Based upon Gene Ontology (GO) annotations [31-33], each of the probe sets represented on the Affymetrix GeneChip^® ^human U133A microarray, having a signal intensity above a background cutoff level, was either assigned to a GO functional group, or else defined as a member of a set excluding that functional group ('non-group members') (see Materials and methods). We asked whether our microarray data might indicate any particular functional groups of genes that were dysregulated in the TS21 samples compared to euploid controls. To address this question, we first performed permutation tests to establish the presence of a signal in the data. Due to the acyclic tree structure of the GO database, with multilevel interconnecting nodes, it is unclear which further permutation test might be performed to optimally define regulated groups. We therefore next applied a *t* test (or Wilcoxon's rank test for groups with only one or two members) to the gene expression data for two groups of probe sets: each given functional group, and the non-group members. This process was then repeated for all the functional groups. We found 1,141 functional groups for the cerebrum, 1,179 functional groups for the cerebellum, 1,126 functional groups for the astrocyte cell lines, and 1,180 functional groups for the heart.

The first 15 functional groups with the smallest *p *values for each tissue/cell type are listed in Tables 5, 6, 7, 8. In particular, the mitochondrion group (n = 417 probe sets) in the fetal cerebrum and heart tissues had the smallest *p *values from our functional group statistical analyses (Tables 5 and 8). Several other groups related to metabolic pathways, such as oxidoreductase activity (n = 299, in the cerebrum), NADH dehydrogenase activity (n = 31, in the cerebrum and heart), and mitochondrial inner membrane (n = 74, in the heart) were also among the most statistically significantly regulated functional groups (Tables 5 and 8).

To establish that there is signal in the data, we also performed permutation tests. For each functional group, a two sample *t* test was carried out, testing for a difference in expression for genes associated with this functional group compared to all other observed gene expression levels. If there were no signal in the data, a random assignment of the expression levels (obtained for example by randomly shuffling the observed expression levels) would yield comparable results. However, the distribution of *p *values obtained from 100 permutation tests (indicated by 100 black lines in the plots) are vastly different from those observed in the original data, indicating that the assumption of no signal in the data was wrong (Additional data files 4 and 5).

For GO functional groups having only one or two genes we applied a Wilcoxon rank test. In each tissue the lowest *p *value ranged from 0.0006 to 0.0726 for the top 20 GO functional groups having only one member, and 0.0001 to 0.1394 for groups having only two members. After correction for multiple comparisons, none of these values is significant (Additional data file 6), suggesting that none of the GO groups comprising one or two members was significantly regulated in TS21 samples from any tissue.

### Confirmation of microarray results

To confirm the altered expression levels of genes detected by microarrays, we performed over 5,600 quantitative real-time PCRs of cDNA derived from total RNA of the fetal samples. We selected a total of 28 genes from those that had shown the most consistent regulation by ANOVA (Tables 1 and 2), including 18 chromosome 21 genes and 10 non-chromosome 21 genes, based upon their abundance, fold regulation, and *p *values. We measured their mRNA levels by quantitative real-time PCR in four tissue/cell types, and compared these levels between TS21 and euploid samples. The hypoxanthine phosphoribosyltransferase (*HPRT*) housekeeping gene was used as a control gene for normalization between samples. Melting curves and gel electrophoresis of PCR products confirmed the identity of the amplification products (data not shown). The directions of dysregulation and fold changes from real-time PCR results were generally consistent with our microarray findings (Tables 9 and 10). Most genes showed increased transcript levels by both microarray and real-time PCR. Two non-chromosome 21 genes, *RRAD *and *ADAMTS8*, were down-regulated in the fetal TS21 heart consistently in microarray and PCR experiments. An example of the results from one real-time PCR experiment for the *ZNF 294 *gene is shown in Additional data file 7.

All microarray data have been submitted to Gene Expression Omnibus (series accession number GSE1397).

## Discussion

The mechanisms by which an extra copy of chromosome 21 produces the phenotype of DS are complex. Epstein and others have postulated that a triplicated chromosome 21 causes a 50% increase in the expression of trisomic genes as a primary dosage effect [5,34]. This primary effect has been observed in several recent studies. We previously measured the expression levels of approximately 15,000 genes in human fetal cerebrum samples, and in astrocytes derived from cerebrum [16]. We observed that RNA transcripts derived from chromosome 21 genes display a dosage-dependent increase in expression. Other groups have reported similar findings in pooled amniotic fluid cells [8] and in whole blood containing multiple cell types [10]. A primary gene dosage effect has also been observed in several mouse models of DS. Ts65Dn [35] and Ts1Cje [36] mice display learning defects and have segmental trisomy of mouse chromosome 16, spanning regions that encode orthologs of about one third to one half of the human chromosome 21 genes. A dosage-dependent increase in the expression of trisomic genes was reported for Ts1Cje [11,12] and Ts65Dn [13,14] mice relative to euploid controls.

In addition to primary gene dosage effects, secondary (downstream) effects on disomic genes are likely to have a major role in aneuploidies in general and DS in particular [5,17,37,38]. However, the nature and extent of such effects in TS21 is controversial [18]. According to one model, trans-acting factors (such as transcription factors) may cause some gene expression changes on chromosomes other than 21, but without a pervasive effect on the transcriptome. Several recent studies support this model. Lyle and colleagues performed quantitative real-time PCR measurements from various tissues of the Ts65Dn mouse, and found changes in the transcript levels of most trisomic genes but zero of 20 disomic genes tested [14]. Similar results were obtained in studies of Ts1Cje mouse brain [11] and cerebellum [12], and in a group of nine tissues in the Ts65Dn mouse [13].

According to a second model, trans-acting factors on chromosome 21 cause a profound disruption of the entire transcriptome. In human cells, FitzPatrick and colleagues [8] reported that genes assigned to chromosome 21 displayed increased transcript levels, but 19 of the 20 most dramatically dysregulated genes did not map to chromosome 21. These results are interpreted as evidence for a mild disomic gene dysregulation [18]. (That study [8] was based on a single initial microarray hybridization. Expression ratios could be measured, but not *p *values to assess the likelihood that those changes occurred by chance.) Tang *et al*. [10], studying blood cells from DS versus control cases, reported that 11 of 56 chromosome 21 genes were expressed at increased levels, but across all chromosomes, 191 genes were up-regulated and 433 genes were down-regulated. In the Ts65Dn mouse, Saran *et al*. [15] measured transcript levels in trisomic and euploid cerebellum, and reported a global destabilization of gene expression, including 922 probes that were significantly, differentially expressed. Even after excluding the 1,532 most regulated probes, they were still able to discriminate trisomic from euploid samples by clustering the remaining gene expression values. This suggests that the expression levels of many thousands of genes are perturbed.

This second model has been supported by other high-throughput approaches. Chrast *et al*. [39], using serial analysis of gene expression, reported 330 tag differences between Ts65Dn and normal mouse brains, about half of which were significantly over-represented. Only three of the 15 genes for which tags were found from the triplicated region of mouse chromosome 16 were overexpressed, so the majority of dysregulated genes were disomic. In another study, results of differential display PCR analysis on neuronal precursor cells derived from the cerebral cortex of a human TS21 fetus showed that *SCG10 *and other genes regulated by the *REST *transcription factor (on chromosome 4) were selectively repressed [40]. We did not observe changes in *REST*-dependent genes as listed in that study (data not shown).

The present study was motivated in part by an attempt to test these models in human tissues, and in particular in tissues that are pathologically affected in DS (cerebellum and heart). Our results support the first model. We measured the expression of thousands of transcripts in cerebellum, cerebrum, and heart, and combined our analyses with those of a previous study of cerebrum and astrocytes [16]. We observed a primary gene dosage effect using both the descriptive statistics approaches of PCA (Figure 1b) and hierarchical clustering (Figure 2a) and the inferential statistics approach of ANOVA (Figures 3 and 4a and Table 1). Using these various approaches, we were unable to distinguish trisomic from euploid samples based on the expression levels of genes assigned to chromosomes other than 21 (Figures 1d, 2b, 3 and 4b). Furthermore, classification using nested cross-validation distinguished trisomic from euploid samples based on chromosome 21 gene expression with extremely high accuracy, but using non-chromosome 21 genes the accuracy was approximately that expected by chance (Tables 3 and 4). As an approach complementary to microarrays, we carried out a systematic study of transcript levels for 28 individual genes by quantitative real-time PCR. These real-time PCR data confirmed our microarrays findings, and they also represent another independent quantitative measurement of RNA transcript levels in the fetal TS21 brain and heart relative to euploid controls.

The two models do not fully reflect the complexity of the trisomic condition; other factors include dosage compensation, the continuum of secondary effects, and tissue specificity. Dosage compensation is a process by which expression levels of sex chromosome-linked genes are rendered equal in males and females of various eukaryotic species [41,42]. Mechanisms include chromosome inactivation, and hypo- as well as hypertranscription of target genes. Dosage compensation for autosomes has been reported for aneuploid conditions in maize and *Drosophila*, organisms for which trisomy is less deleterious than in humans [37,38,43,44]. Dosage compensation also likely occurs in DS, such that some trisomic chromosome 21 genes are not expressed at elevated levels [5].

In each of the four tissue/cell types we studied, approximately one third of all chromosome 21 genes was expressed, and of these, only a subset of transcripts was expressed at higher levels relative to euploid controls (Figure 4a and Table 1). Our study included a sufficient sample size to perform ANOVA, as well as quantitative real-time PCR (Table 9), and thus we defined several dozen specific chromosome 21 genes that are dysregulated. Those chromosome 21 genes that were expressed but not regulated may have been subject to dosage compensation. A variety of other human studies, including our previous work [16], lacked sufficient samples and/or microarray replicates to define significantly regulated genes based on a *t* test or ANOVA with a correction for multiple comparisons [8,9,19,20].

The secondary effects of TS21 may include either limited or extensive changes to non-chromosome 21 genes, but these alternatives represent extremes of a continuum. Most autosomal aneuploidies are not compatible with life, and each of the most common syndromes (trisomies 13, 18, and 21) likely causes distinct secondary effects based on the particular transcription factors, modifiers of chromatin, or other gene products at dosage imbalance. We identified the significant regulation of at least one transcription factor (*ZNF294*; Table 1). The varying results for secondary transcriptional effects reported for human TS21 versus mouse Ts65Dn and Ts1Cje models could, to some extent, reflect differences in the particular transcriptional regulators that are present at dosage imbalance in each system, as well as other factors such as differences in dosage compensation. Another variable is the particular developmental stage being studied, which could have a dramatic effect on both primary and secondary transcriptional effects of trisomy.

The tissue specificity of gene expression in the aneuploid state represents an additional level of complexity. For the four tissue and cell types we studied, RNA transcripts from chromosome 21 genes were significantly elevated. However, both of the ANOVA results (Table 2) and real-time PCR assays (Table 10) indicated that there were tissue-specific changes in transcript levels for individual genes on other chromosomes. These include those genes predominantly expressed in the heart but not in the brain, even though the primary genetic insults in all these different tissue or cell types were all an extra copy of chromosome 21. Our analyses on groups of genes that are functionally related also suggested similar region-specific differences in transcription across multiple tissue and cell types (Tables 5, 6, 7, 8). This tissue specificity was also noted in recent mouse models of DS [13,14]. Our study has further significance because we have identified significantly regulated transcripts in affected human tissues. Thus, while template availability results in increased production of RNA transcripts, factors that regulate tissue-specific gene expression have a major role in controlling which specific transcripts are expressed at dosage imbalance.

Among the significantly regulated genes from ANOVA (Tables 1 and 2), several encode proteins that have roles in mitochondrial function: *ATP5O *and *ATP5J *(two genes encoding subunits of ATP synthase), and mitochondrial ribosomal protein L39 (*MRPL39*). Their expression levels were increased based on our microarray experiments (Table 1) and subsequent real-time PCR (Table 9). Additionally, various mitochondrion-related functional groups were significantly regulated (see Results and Tables 5 and 8). Abnormal regulation of these transcripts and functional groups could contribute to the impaired mitochondrial function that has been observed in DS [45].

The type VI collagen genes on chromosome 21 have been thought to be involved in the congenital heart defect phenotype in DS [46,47]. Consistent with this finding, our microarray study indicated that the type VI collagen alpha 1 gene (*COL6A1*) was one of the most regulated genes (Table 1). Furthermore, six of the non-chromosome 21 genes are associated with cardiac muscle, such as myomesin, myoglobin, and calsequestrin 2 (Table 2). They are all up-regulated (1.34 to 4.17-fold increase) in TS21 fetal heart tissues that consisted of primarily the pulmonary, tricuspid, aortic and mitral valves, ventricular septum, atrial septum, atrioventricular valve, and some surrounding tissues, which are regions in the heart most commonly affected in DS. Among all AVSD cases, 43% are associated with DS [26]. In particular, the ventricular inlet septum has been reported to be underdeveloped at all stages between 5 and 16 gestational weeks, among other developmental abnormalities [48]. We postulate that the up-regulation of genes related to cardiac muscle may be a compensatory response for developmental defects due to trisomy in the DS hearts. For example, in complete AVSD, deficiency of the atrial septum, ventricular septum, and atrioventricular valve result in abnormal communication between the four cardiac chambers, allowing oxygen-rich blood to regurgitate or leak backwards from the left ventricle into the left or right atrium, and back to the lungs again. This causes more work for the heart. With AVSDs, the heart can hypertrophy, as we observed in the TS21 fetal hearts (data not shown). It is possible that the TS21 heart up-regulates muscle-related genes as a secondary effect of the triplication of the entire chromosome 21 or of individual genes. Of 79 genes defined by Barlow *et al*. [29] as forming a critical region on chromosome 21 for congenital heart disease, seven had increased expression in our study (*SH3BGR*, *CSTB*, *PFKL*, *PDXK*, *TMEM1*, C21orf33, *WRB*) (Tables 1 and 9). Although during our dissection we discarded the surrounding muscle tissue wherever it was possible, we cannot eliminate the possibility that our dissection of fetal heart tissue containing predominantly valve and canal regions might have included more muscle tissue in the TS21 cases than in the controls.

## Conclusion

In the present study we report dosage-dependent transcription in human fetal tissues that are pathologically affected in DS. We also identified individual differentially expressed genes based on criteria of statistical significance. For 28 of these genes, we confirmed the regulation by quantitative real-time PCR. The data indicate a primary gene dosage effect in which, in each tissue tested, a group of genes assigned to chromosome 21 were expressed at higher levels relative to euploid controls. Furthermore, while we observed changes in some transcripts derived from non-chromosome 21 genes, our data do not support a model in which there is large-scale disruption of the transcriptome.

Our data indicated that there were tissue- and cell-specific changes of gene expression in TS21 during fetal development. The functional groups indicated by statistical analyses on our microarray data provided initial indications of possible biological pathways affected by TS21. However, the relationship between levels of RNA and the corresponding protein products is at present unknown. As a next step to understand how the changes at the transcript levels lead to DS phenotypes, it is important to analyze the translational machinery by characterization of TS21 protein profiles.

## Materials and methods

### Microarray sample dissection and RNA isolation

All human tissues were obtained from the Brain and Tissue Bank for Developmental Disorders at the University of Maryland with informed consent using Institutional Review Board-approved protocols. Diagnoses, gender, race, and other information is provided in Additional data file 1. Three TS21 and three age- and gender-matched control cerebella were dissected from frozen fetal brains. For the two TS21 and two matched control frozen fetal heart tissues, the regions that contain primarily the pulmonary, tricuspid, aortic and mitral valves, ventricular septum, atrial septum, atrioventricular valve, and some surrounding tissues were dissected. Wherever possible, the peripheral heart muscle tissue was removed to minimize the amount of RNA from muscle tissue. Total RNA was extracted from frozen tissues using RNeasy^® ^Midi Kit (Qiagen, Valencia, CA, USA) according to the manufacturer's instructions. The quantity and purity of RNA were confirmed by spectrophotometry and agarose gel electrophoresis.

### Gene expression data acquisition and pre-processing

Gene expression data were obtained using Affymetrix U133A GeneChip^® ^with standard protocols [49] at the Johns Hopkins Microarray Core Facility. Raw data from U133A GeneChips were processed using both Affymetrix Microarray Suite version 5.0 (MAS5) software and robust multi-chip analysis (RMA) normalization (R version 1.7.1) from BioConductor [50]. The results using either MAS5 (described below) or RMA (data not shown) were very similar, and we did not have a compelling reason to favor one method over the other for this study. In Affymetrix MAS5, signal is calculated using the One-Step Tukey's Biweight Estimate which yields a robust weighted mean. The U133A GeneChip contains a total of 22,284 probes. We removed 2,023 Affymetrix bacterial and housekeeping control probes and probes that do not map to a known chromosomal location. This resulted in 20,261 probes. The data were further subdivided into probes that code for genes assigned to chromosome 21 (n = 253) and probes that code for genes assigned to all other chromosomes (n = 20,008) or for each chromosome (Additional data file 2). The Present/Absent description of probes by MAS5 software was not used in our analyses. Data from astrocytes and cerebrum were previously published [16] and were reanalyzed in this study.

### Expression data analysis: exploratory analyses

Exploratory analyses using PCA [51] and hierarchical clustering were performed using Partek^® ^software [52]. All probes (n = 253 from chromosome 21 and n = 20,008 from other chromosomes) were used for these analyses. For PCA, we used the covariance dispersion matrix option. The ellipses were drawn at three standard deviations around the centroid of the samples for each of the four tissues (Figure 1). Hierarchical clustering was performed on the 25 tissue samples based on chromosome 21 genes and again based on non-chromosome 21 genes. In each case the Euclidean distance was used, although similar results were achieved using other measures of dissimilarity. Cluster merging was performed using average linkage. The horizontal axes of the dendrograms (Figure 2) correspond to dissimilarity.

### Expression data analysis: statistical testing

A mixed-model ANOVA was used to detect differential expression at individual gene level and at the chromosome level. The ANOVA model was chosen to partition subject-to-subject, tissue, and disease type variability from variability due to biological and experimental noise. ANOVA was performed using Partek^® ^software [52]. The following linear mixed model (equation 1) was used to detect differential expression on a gene-by-gene basis:

*y*_*ijk *_= *D*_*i *_+ *T*_*j *_+ *DT*_*ij *_+ *S*(*D*)_*ik *_+ *ε*_*ijk*_

where *y*_*ijk *_is the expression of the gene for *i*th disease type, *j*th tissue, and *k*th subject. The symbols *D*, *T*, *DT*, and *S*(*D*) represent effects due to disease, tissue, disease-by-tissue interaction, and subject-nested-within-disease, respectively. The error for each gene for sample *ijk *is designated as *ε*_*ijk*_. Tissue and disease are fixed effects and subject is a random effect in the mixed model. The average R^2 ^value for genes assigned to chromosome 21 was 0.760 and for all genes assigned to other chromosomes was 0.757. This indicates that approximately 76% of the variance in the data was explained by the ANOVA model of equation 1.

To test chromosomes for differential expression, for each of the 25 tissue samples we first averaged all genes from a particular chromosome. For example, a total of 253 expression values (corresponding to 253 probe sets) assigned to chromosome 21 were averaged. This resulted in 23 values for each tissue sample, with each value representing the average expression of all genes on each chromosome for that tissue sample (chromosome X and Y were combined). The linear model of equation 1 was used to test for differential expression between TS21 and euploid controls to test our hypothesis that some chromosomes may show overall differential expression between TS21 and control groups. In each case, the Benjamini-Hochberg step-up FDR [30] was applied to determine the list of genes deemed to be statistically significant.

### Expression data analysis: class prediction

We investigated the ability to classify tissue samples as TS21 or euploid controls based on the expression of genes assigned to chromosome 21, or to other genes. We used Partek^® ^software for these analyses. Detailed methods are available in Additional data file 3 [53]. Briefly, our classification tests employed a nested leave-one-subject-out cross-validation step that was carried out in three parts: gene selection, selection of an optimal classifier, and estimation of classification accuracy. For gene selection (variable selection) we used ANOVA, and varied the number of predictor genes. For selection of an optimal classifier, the methods that we employed were K-Nearest Neighbor [54], Nearest 'Shrunken' Centroid [55], and Discriminant Analysis. For estimation of the classification accuracy, nested cross-validation was performed (see Additional data file 3). The nested cross-validation is performed using an 'outer' cross-validation that was used to obtain accuracy estimates, and a nested, 'inner' cross-validation that was used to select genes and tune classifier parameters.

### Expression data analysis: functional group testing

Most of the probe sets on the Affymetrix GeneChip^® ^human U133A microarray can be assigned to one or more functional groups with a unique ID number based upon GO annotations [31-33]. GO IDs are organized in a tree-like structure via parent-child relationships. The top level has only one group: 'Gene_Ontology', which is then sub-divided into three groups at the second level, including biological_process, cellular_component, and molecular_function. To assess the statistical significance of gene expression differences in distinct functional groups, we implemented a novel *t* test procedure that we named a 5T analysis (tree-travel, transform, *t* test). This algorithm differs from web-based tools such as GoMiner [56], FatiGO [57], GO:TermFinder [58], or GOTree Machine [59], which define genes as either regulated or not, and employ a Fisher's exact test or hypergeometric distribution analysis. Under the usual assumptions, namely independence and normality of the error, a *t* test offers more power than a test with a dichotomized outcome. Our algorithm also differs from methods such as MAPPFinder [60] that assess the significance of a user-defined, predetermined set of genes of interest.

A detailed description of the 5T method is presented in Additional data file 3 [53]. Briefly, the first step is tree-travel: for each probe set, we parsed its GO annotations, and generated a list of functional groups located in the top six levels of GO tree structure. In the transform step, we generated a list of probe sets assigned to a functional group and a list of probe sets not assigned to this functional group ('non-group members'). In the *t* test step, for each functional group with three or more members in a tissue/cell type, we performed a *t* test on this group and non-group members using log ratio gene expression values. The process was repeated for all the functional groups. We then sorted all the functional groups in a tissue/cell type based on their *p *values from the *t* tests. To avoid discarding potentially useful information, we also performed Wilcoxon's rank test to assess the statistical significance of differentially regulated functional groups having only one or two members.

We also applied an alternative statistical test to the data based upon a permutation principle. We started with a list of probe sets assigned to a particular functional group. We then randomly selected an equal number of probe sets from all probe sets on the microarray and calculated the mean log ratio values. This random selection was repeated 100 times. The average of the mean log ratio values was calculated, and compared to the mean log ratio value of that particular functional group. The permutation test was performed on all functional groups.

### Quantitative real-time PCR

Total RNA was isolated from frozen tissues or astrocytes using RNeasy^® ^Midi Kit (Qiagen) and followed by cDNA synthesis using Invitrogen SuperScript™ First-Strand System for RT-PCR (Invitrogen Life Technologies, Carlsbad, CA, USA). Quantitative real-time PCR was performed by a 7900HT Sequence Detector System (Applied Biosystems, Foster City, CA, USA) or LightCycler (Roche Molecular Biochemicals, Indianapolis, IN, USA). Primer sequences are described in Additional data file 8. The expression level of the *HPRT *housekeeping gene was used for normalization. Detailed methods are provided in Additional data file 3 [53].

## Additional data files

The following additional data are included with the online version of this article. Additional data file 1 is a word document entitled 'Information on samples used in microarray studies'. It lists information on 25 samples such as race, gender, and postmortem interval. Additional data file 2 is a word document entitled 'Results of test for whether individual genes assigned to any chromosome were differentially expressed in TS21 relative to euploid samples'. This table describes FDR results shown for each individual chromosome. Additional data file 3 is a word document entitled 'Additional methods'. This file provides detailed methods for the following topics: Expression data analysis: class prediction; Error estimation using nested cross-validation; Selection of predictor genes for classification; Expression data analysis: functional group testing; and Quantitative real-time PCR. The functional group testing section includes the description of a novel algorithm for functional group analyses. Additional data file 4 is a word document that provides figure legends for the Additional data file 5 and 7 figures. Additional data file 5 is an EPS file entitled 'Permutation test on GO functional groups'. This figure shows the results of permutation tests, providing evidence that the functional groups we identified are likely to have been identified with a probability far greater than is expected by chance (as determined by a series of random permutations of the data). Additional data file 6 is a word document entitled 'Results of Wilcoxon rank test for analysis of functional group regulation'. This table provides results of a Wilcoxon rank test that is appropriate for functional groups having a small size. Additional data file 7 is a tif file entitled 'Relative amounts of ZNF294 transcripts present in the fetal TS21 and euploid cerebrum samples detected by quantitative real-time PCR'. This figure shows a typical quantitative real-time PCR result, in which the level of a transcript is significantly up-regulated in a trisomic sample. Additional data file 8 is a word document entitled 'Primer sequences and other information of the quantitative real-time PCR experiments'. This table includes oligonucleotide sequences.

## Supplementary Material

Additional data file 1 Lists information on 25 samples such as race, gender, and postmortem interval. Click here for file

Additional data file 2 This table describes FDR results shown for each individual chromosome. Click here for file

Additional data file 3 Detailed methods for the following topics: Expression data analysis: class prediction; Error estimation using nested cross-validation; Selection of predictor genes for classification; Expression data analysis: functional group testing; and Quantitative real-time PCR. The functional group testing section includes the description of a novel algorithm for functional group analyses. Click here for file

Additional data file 4 Figure legends for the Additional data file 5 and 7 figures. Click here for file

Additional data file 5 This figure shows the results of permutation tests, providing evidence that the functional groups we identified are likely to have been identified with a probability far greater than is expected by chance (as determined by a series of random permutations of the data). Click here for file

Additional data file 6 This table provides results of a Wilcoxon rank test which is appropriate for functional groups having a small size. Click here for file

Additional data file 7 This figure shows a typical quantitative real-time PCR result, in which the level of a transcript is significantly up-regulated in a trisomic sample. Click here for file

Additional data file 8 This table includes oligonucleotide sequences. Click here for file

## Figures and Tables

**Table 1:** Most consistently dysregulated chromosome 21 genes based on their *p*-values from ANOVA and after 5% false discovery rate cut-off

Gene name	Accession number	Chromosome number	*p *value (ANOVA)	Cerebrum		Cerebellum		Astrocyte		Heart	
				
				Control	TS21	Control	TS21	Control	TS21	Control	TS21
Pituitary tumor-transforming 1 interacting protein (*PTTG1IP*)	NM_004339	21	1.50E-07	582.6	888.1	830.9	1176.9	2355.5	3896.0	1153.0	2003.5
ATP synthase, H+ transporting, mitochondrial F1 complex, O subunit (*ATP5O*)	NM_001697	21	5.11E-07	1509.0	2553.5	1331.5	2327.1	1552.9	2086.3	2375.0	4002.1
SH3 domain binding glutamic acid-rich protein (*SH3BGR*)	NM_007341	21	7.12E-07	20.5	44.5	21.2	48.4	38.2	130.2	606.8	1937.5
ATP synthase, H+ transporting, mitochondrial F0 complex, subunit F6 (*ATP5J*)	NM_001685	21	2.47E-06	624.4	1148.8	723.1	1013.6	881.3	1331.5	916.4	2046.7
Down syndrome critical region gene 3 (*DSCR3*)	NM_006052	21	1.44E-05	51.7	94.3	49.8	92.6	49.7	169.0	72.9	71.1
Chromosome 21 segment HS21C048, zinc finger protein 294 (*ZNF294*)	NM_015565	21	3.39E-05	165.7	283.0	161.6	228.9	78.6	127.8	107.5	178.0
Superoxide dismutase 1 (*SOD1*)	NM_000454	21	5.62E-05	1176.2	2493.4	1816.7	2860.4	2482.7	3853.6	1789.7	3110.8
ATP synthase, H+ transporting, mitochondrial F1 complex, O subunit (*ATP5O*)	NM_001697	21	6.94E-05	203.7	335.9	219.1	342.7	124.5	258.4	342.4	521.4
Cystatin B (stefin B) (*CSTB*)	NM_000100	21	7.75E-05	412	695.0	584.6	868.9	855.1	1007.3	797.4	1034.7
Phosphofructokinase, liver (*PFKL*)	BC006422	21	1.93E-04	411	476.9	255.8	492.1	247.3	397.9	390.0	433.1
Pyridoxal (pyridoxine, vitamin B6) kinase (*PDXK*)	NM_003681	21	2.82E-04	50.3	137.4	70.1	149.4	118.4	261.6	96.6	139.3
Collagen, type VI, alpha 1 (*COL6A1*)	AA292373	21	5.04E-04	559.4	963.1	1019	1417	573.7	834.4	3003.5	4177.7
Transmembrane protein 1 (*TMEM1*)	U61500	21	5.25E-04	68.4	83.6	45.0	90.8	34.5	88.5	6.6	62.8
Ubiquitin specific protease 16 (*USP16*)	NM_006447	21	5.33E-04	189.8	318.8	223.1	306.5	272.5	513.4	180.0	320
SMT3 suppressor of mif two 3 homolog 1 (yeast) (*SMT3H1*)	NM_006936	21	6.27E-04	704.0	1181.5	823.4	1233.1	698.7	1092.9	484.6	676.5
SON DNA binding protein (*SON*)	X63071	21	7.28E-04	701.5	975.7	807.4	870.3	781.2	1181.3	761.7	924.7
Mitochondrial ribosomal protein L39 (*MRPL39*)	NM_017446	21	7.48E-04	195.2	281.5	256.7	266.2	250.6	310.1	274.1	385.9
Interferon gamma receptor 2 (*IFNGR2*)	NM_005534	21	8.16E-04	553.5	754.3	507.5	692.0	881.2	1307.9	639.5	811.15
Human homolog of ES1 (zebrafish) protein (C21orf33)	D86062	21	1.02E-03	175.5	260.5	163.5	280.1	190.0	202.1	188.4	374.7
Chaperonin containing TCP1, subunit 8 (theta) (*CCT8*)	NM_006585	21	1.45E-03	1098	1520.4	743.6	956.3	619.0	1200.8	615.1	1089.8
Chromosome 21 open reading frame 108 (C21orf108)	AI803485	21	1.53E-03	52.5	101.9	61.9	91.8	60.7	105.4	25.6	71.3
Tryptophan rich basic protein (*WRB*)	NM_004627	21	2.18E-03	759.6	1439.2	926.4	1182.4	728.6	1336.5	291.9	566.5
SMT3 suppressor of mif two 3 homolog 1 (yeast) (*SMT3H1*)	BG338532	21	3.15E-03	204.0	274.6	186.6	294.2	252.2	352.2	157.3	263.7
HMT1 hnRNP methyl-transferase-like 1 (*HRMT1L1*)	NM_001535	21	3.62E-03	670.0	920.5	584.2	843.2	489.1	471.6	363.0	525.2
Human homolog of ES1 (zebrafish) protein (C21orf33)	NM_004649	21	4.00E-03	491.8	818.2	589.7	918.9	455.9	665.6	713.3	1039.4
Stress 70 protein chaperone, microsome-associated, 60 kDa (*STCH*)	AI718418	21	4.43E-03	276.2	477.5	289	308.5	418.2	738.6	59.0	111.4

The average expression values are for the probe sets corresponding to the genes (from MAS5 software). Two genes (ATP5O and C21orf33) each have two probe sets on this list. TS21, trisomy 21.

**Table 2:** Most consistently dysregulated non-chromosome 21 genes based on their *p *values from ANOVA and after 5% false discovery rate cut-off

Gene name	Accession number	Chromosome number	*p *value (ANOVA)	Cerebrum		Cerebellum		Astrocyte		Heart	
				
				Control	TS21	Control	TS21	Control	TS21	Control	TS21
Hypermethylated in cancer 1 (*HIC1*)	NM_006497	17	2.33E-08	6.5	1.9	4.8	3.8	4.6	2.0	41.3	5.8
Myomesin 1 (skelemin) (185 kDa) (*MYOM1*)	NM_003803	18	8.82E-08	37.8	23.3	45.0	52.6	13.6	9.8	930.1	1302.5
Myoglobin (*MB*)	NM_005368	22	1.09E-07	103.5	85.5	90.2	142.8	72.9	61.1	7392.9	12099.8
Calsequestrin 2 (cardiac muscle) (*CASQ2*)	NM_001232	1	1.56E-07	17.7	9.3	14.1	19.5	14.4	14.3	2341.5	3868.7
Ras-related associated with diabetes (*RRAD*)	NM_004165	16	5.06E-06	4.5	4.2	13.3	9.8	45.8	36.6	1907.1	932.0
Troponin I, cardiac (*TNNI3*)	NM_000363	19	5.90E-06	49.0	44.1	44.6	71.2	31.1	25.2	2942.4	4757.2
Insulin-like growth factor binding protein 7 (*IGFBP7*)	NM_001553	4	1.12E-05	223.8	314.7	741.5	519.4	2418.6	4205.6	743.8	1137.2
Actin, alpha 1, skeletal muscle (*ACTA1*)	NM_001100	1	1.20E-05	38.6	38.5	33.7	47.6	55.9	138.1	553.4	2310.0
Calcineurin-binding protein calsarcin-1 (*MYOZ2*)	NM_016599	4	1.22E-05	4.9	6.3	7.6	20.2	4.7	3.0	1742.3	2592.5
Teratocarcinoma-derived growth factor 1 (*TDGF1*)	NM_003212	3	1.95E-05	10.6	11.8	8.2	9.9	31.1	20.6	11.3	187.9
Tenomodulin protein (*TNMD*)	NM_022144	X	2.24E-05	7.2	5.4	10.0	6.4	5.8	4.8	23.6	103.0
Olfactory receptor, family 7, subfamily E, member 12 pseudogene (OR7E12P)	AA459867	13	2.51E-05	115.4	88.7	149.1	87.6	144.8	116.1	215.1	58.4
Cardiac troponin T2 (*TNNT2*)	X79857	1	2.56E-05	47.4	39.9	47.4	45.7	44.6	32.6	3710.3	4965.9
A disintegrin-like and metalloprotease (reprolysin type) with thrombospondin type 1 motif, 8 (*ADAMTS8*)	NM_007037	11	3.21E-05	13.0	11.5	14.6	15.5	15.1	11.4	282.8	154.7

The average expression values are for the probe sets corresponding to the genes (from MAS5 software). TS21, trisomy 21.

**Table 3:** Nested cross-validation results using chromosome 21 genes

Pass	Number of samples	Best inner C-V score (% correct)	Number of tied models	Outer C-V score (% correct)
Subject 1	3	100.00% (22/22)	116	100.00%
Subject 2	2	100.00% (23/23)	160	100.00%
Subject 3	4	100.00% (21/21)	119	100.00%
Subject 4	4	100.00% (21/21)	142	99.82%
Subject 5	4	100.00% (21/21)	107	100.00%
Subject 6	1	100.00% (24/24)	131	100.00%
Subject 7	4	100.00% (21/21)	247	99.60%
Subject 8	1	100.00% (24/24)	186	100.00%
Subject 9	1	100.00% (24/24)	107	100.00%
Subject 10	1	100.00% (24/24)	212	100.00%
			Accuracy estimate	99.91%

The model space parameters are as follows: Gene selection: ANOVA; Number of genes: 1, 3, 5, ..., 251, 253; Classifier 1: K-Nearest Neighbor (KNN); Number of neighbors (K): 1, 3, 5; Similarity measures: Euclidean distance, Pearson's correlation, Absolute value (also known as 'City block'); Classifier 2: Nearest Centroid, Prior probability: Equal; Classifier 3: Discriminant Analysis, Discriminant functions: Linear, Quadratic, Prior probability: Equal.

**Table 4:** Nested cross-validation results using non-chromosome 21 genes

Pass	Number of samples	Best inner C-V score (% correct)	Number of tied models	Outer C-V score (% correct)
Subject 1	3	68.18% (15/22)	12	13.89%
Subject 2	2	69.57% (16/23)	92	1.63%
Subject 3	4	71.43% (15/21)	3	50.00%
Subject 4	4	66.67% (14/21)	3	75.00%
Subject 5	4	76.19% (16/21)	13	25.00%
Subject 6	1	75.00% (18/24)	18	0.00%
Subject 7	4	57.14% (12/21)	4	68.75%
Subject 8	1	70.83% (17/24)	1	100.00%
Subject 9	1	70.83% (17/24)	24	95.83%
Subject 10	1	66.67% (16/24)	1	100.00%
			Accuracy estimate	48.63%

The classifier space evaluated was the same as the one used in the chromosome 21 test (Table 3).

**Table 5:** Most statistically significantly regulated functional groups in the fetal cerebrum tissues based on their *p *values from *t* tests

GO group	GO identifier	Number of probe sets	*p *value	Mean of GO group	Mean of non-group members
Mitochondrion	GO:0005739	417	1.41E-14	0.03	0
Monovalent inorganic cation transporter activity	GO:0015077	86	3.72E-09	0.05	0
Nucleobase, nucleoside, nucleotide and nucleic acid metabolism	GO:0006139	1495	2.00E-07	-0.02	0
Nucleus	GO:0005634	2072	5.50E-07	-0.01	0
Nucleic acid binding	GO:0003676	485	1.23E-06	-0.02	0
Oxidoreductase activity	GO:0016491	299	3.31E-06	0.02	0
NADH dehydrogenase activity	GO:0003954	31	3.80E-06	0.06	0
DNA binding	GO:0003677	1071	1.35E-05	-0.02	0
Cytochrome-c oxidase activity	GO:0004129	25	8.51E-05	0.05	0
RNA binding	GO:0003723	466	1.48E-04	-0.02	0
Transcription factor activity	GO:0003700	558	2.40E-04	-0.02	0
Amine metabolism	GO:0009308	147	3.47E-04	0.03	0
RNA metabolism	GO:0016070	273	7.76E-04	-0.02	0
Heterotrimeric G-protein GTPase, alpha-subunit	GO:0000263	8	8.51E-04	0.04	0
Helicase activity	GO:0004386	27	1.15E-03	-0.05	0

The Gene Ontology (GO) database was used to assign a probe set to a functional group. There were 736 functional groups tested for the cerebrum tissue. The first 12 functional groups with the smallest *p* values are listed here. The mean of log ratios between trisomy 21 and euploid controls for each functional group was compared to that for the group of remaining probe sets not assigned to that functional group ('non-group members'). After one type of multiple test comparison correction, the cut-off level for statistical significance was 6.79E-05 (assigned by dividing 0.05 by the number of functional groups, 736).

**Table 6:** Most statistically significantly regulated functional groups in the fetal cerebellum tissues based on their *p *values from *t* tests

GO group	GO identifier	Number of probe sets	*p *value	Mean of GO group	Mean of non-group members
Integral to plasma membrane	GO:0005887	933	1.26E-11	0.03	0
RNA binding	GO:0003723	469	6.31E-11	-0.02	0.01
Structural constituent of ribosome	GO:0003735	212	1.12E-08	-0.03	0.01
G-protein coupled receptor activity	GO:0004930	212	1.29E-08	0.06	0
Transmission of nerve impulse	GO:0019226	170	3.31E-08	0.06	0
Nucleus	GO:0005634	2194	3.55E-08	-0.01	0.01
Cell surface receptor linked signal transduction	GO:0007166	592	2.24E-07	0.03	0
Ribosome	GO:0005840	147	4.68E-07	-0.03	0.01
Defense response	GO:0006952	430	1.07E-06	0.04	0
Nucleobase, nucleoside, nucleotide and nucleic acid metabolism	GO:0006139	1596	5.37E-06	-0.01	0.01
Neurogenesis	GO:0007399	337	9.55E-06	0.04	0
Eukaryotic translation initiation factor 4 complex	GO:0008304	13	2.51E-05	-0.05	0.01
RNA metabolism	GO:0016070	286	4.57E-05	-0.02	0.01
GABA receptor activity	GO:0016917	15	6.46E-05	0.1	0.01
DNA binding	GO:0003677	1126	7.24E-05	-0.01	0.01

There were 764 functional groups tested. The first 12 functional groups with the smallest *p* values are listed here. The mean of log ratios between TS21 and euploid controls for each functional group was compared to that for the group of remaining probe sets not assigned to that functional group ("non-group members"). After one type of multiple test comparison correction, the cutoff level for statistical significance was 6.54E-05 (assigned by dividing 0.05 by the number of functional groups, 764).

**Table 7:** The most statistically significantly regulated functional groups in the fetal astrocyte cell lines based on their *p* values from *t* tests

GO group	GO identifier	Number of probe sets	*p *value	Mean of GO group	Mean of non-group members
Collagen	GO:0005581	15	7.76E-07	0.33	-0.01
Endoplasmic reticulum	GO:0005783	307	8.91E-07	0.03	-0.02
Fibrillar collagen	GO:0005583	14	5.01E-06	0.29	-0.01
Intracellular non-membrane-bound organelle	GO:0043232	122	7.94E-06	-0.06	-0.01
G-protein coupled receptor activity	GO:0004930	141	1.26E-05	-0.09	-0.01
Gametogenesis	GO:0007276	66	2.45E-05	-0.08	-0.01
Integral to plasma membrane	GO:0005887	734	2.57E-05	-0.04	-0.01
Intramolecular oxidoreductase activity, interconverting keto- and enol-groups	GO:0016862	11	2.19E-04	0.04	-0.01
Carbohydrate transport	GO:0008643	16	2.40E-04	0.1	-0.01
DNA metabolism	GO:0006259	309	3.98E-04	-0.04	-0.01
Extracellular matrix (sensu Metazoa)	GO:0005578	120	6.76E-04	0.09	-0.02
Muscle development	GO:0007517	82	6.76E-04	0.06	-0.01
Defense response	GO:0006952	381	8.51E-04	-0.04	-0.01
Cell adhesion	GO:0007155	227	8.91E-04	0.04	-0.01
MHC class II receptor activity	GO:0045012	12	9.12E-04	-0.12	-0.01

There were 734 functional groups tested. The first 12 functional groups with the smallest *p* values are listed here. The mean of log ratios between trisomy 21 and euploid controls for each functional group was compared to that for the group of remaining probe sets not assigned to that functional group ('non-group members'). After one type of multiple test comparison correction, the cut-off level for statistical significance was 6.81E-05 (assigned by dividing 0.05 by the number of functional groups, 734).

**Table 8:** Most statistically significantly regulated functional groups in the fetal heart tissues based on their *p* values from *t* tests

GO group	GO identifier	Number of probe sets	*p *value	Mean of GO group	Mean of non-group members
Mitochondrion	GO:0005739	446	6.61E-16	0.04	-0.01
Monovalent inorganic cation transporter activity	GO:0015077	86	4.27E-10	0.07	-0.01
Defense response	GO:0006952	485	2.29E-08	-0.06	-0.01
NADH dehydrogenase activity	GO:0003954	31	5.25E-06	0.07	-0.01
Intracellular transport	GO:0046907	428	2.34E-05	0.02	-0.01
Cell-cell signaling	GO:0007267	201	1.02E-04	-0.07	-0.01
Mitochondrial inner membrane	GO:0005743	74	1.66E-04	0.04	-0.01
Cell surface receptor linked signal transduction	GO:0007166	594	1.74E-04	-0.04	-0.01
Integral to plasma membrane	GO:0005887	948	1.95E-04	-0.03	-0.01
Extracellular region	GO:0005576	215	2.40E-04	-0.06	-0.01
Membrane fusion	GO:0006944	14	4.68E-04	0.06	-0.01
DNA metabolism	GO:0006259	322	8.13E-04	0.02	-0.01
Regulation of muscle contraction	GO:0006937	28	9.33E-04	0.08	-0.01
Single-stranded DNA binding	GO:0003697	45	1.38E-03	0.06	-0.01
Electron carrier activity	GO:0009055	12	1.41E-03	0.08	-0.01

There were 769 functional groups tested. The first 12 functional groups with the smallest *p *values are listed here. The mean of log ratios between trisomy 21 and euploid controls for each functional group was compared to that for the group of remaining probe sets not assigned to that functional group ('non-group members'). After one type of multiple test comparison correction, the cut-off level for statistical significance was 6.50E-05 (assigned by dividing 0.05 by the number of functional groups, 769).

**Table 9:** Quantitative real-time PCR results for selected chromosome 21 genes in Table 1

Gene name	Chromosome	*p *value	Cerebrum		Cerebellum		Astrocyte		Heart	
			
			Microarray	qPCR	Microarray	qPCR	Microarray	qPCR	Microarray	qPCR
Pituitary tumor-transforming 1 interacting protein (*PTTG1IP*)	21	1.50E-07	1.52	3.85 ± 0.38	1.42	1.46 ± 0.62	1.65	2.42 ± 1.16	1.74	1.41 ± 0.16
ATP synthase, H+ transporting, mitochondrial F1 complex, O subunit (*ATP5O*)	21	5.11E-07	1.69	1.52 ± 0.20	1.75	0.91 ± 0.16	1.34	1.48 ± 0.72	1.69	3.52 ± 1.22
ATP synthase, H+ transporting, mitochondrial F0 complex, subunit F6 (*ATP5J*)	21	2.47E-06	1.84	1.25 ± 0.14	1.40	1.19 ± 0.12	1.51	1.73 ± 0.78	2.23	5.19 ± 1.60
Down syndrome critical region gene 3 (*DSCR3*)	21	1.44E-05	1.82	2.76 ± 0.44	1.86	0.79 ± 0.38	3.40	2.50 ± 0.62	0.98	2.06 ± 0.06
Chromosome 21 segment HS21C048, zinc finger protein 294 (*ZNF294*)	21	3.39E-05	1.71	2.58 ± 0.24	1.42	1.30 ± 0.42	1.63	1.60 ± 0.24	1.66	1.51 ± 0.14
Superoxide dismutase 1 (*SOD1*)	21	5.62E-05	2.12	1.72 ± 0.24	1.57	1.44 ± 0.12	1.55	2.75 ± 1.37	1.74	2.86 ± 1.52
Cystatin B (stefin B) (*CSTB*)	21	7.75E-05	1.69	1.21 ± 0.13	1.49	1.21 ± 0.14	1.18	1.92 ± 0.56	1.30	1.88 ± 0.07
Phosphofructokinase, liver (*PFKL*)	21	1.93E-04	1.16	2.17 ± 0.14	1.92	1.35 ± 0.08	1.61	2.07 ± 0.44	1.11	2.23 ± 0.55
Pyridoxal (pyridoxine, vitamin B6) kinase (*PDXK*)	21	2.82E-04	2.73	2.99 ± 0.40	2.13	1.79 ± 0.20	2.21	2.18 ± 1.01	1.44	1.75 ± 0.09
Collagen, type VI, alpha 1 (*COL6A1*)	21	5.04E-04	1.72	3.16 ± 0.24	1.39	0.75 ± 0.06	1.45	2.57 ± 1.28	1.39	1.97 ± 0.76
Ubiquitin specific protease 16 (*USP16*)	21	5.33E-04	1.80	2.75 ± 0.48	1.30	1.33 ± 0.36	1.67	1.70 ± 0.50	1.67	3.40 ± 1.54
SMT3 suppressor of mif two 3 homolog 1 (yeast) (*SMT3H1*)	21	6.27E-04	1.68	1.66 ± 0.42	1.50	0.93 ± 0.08	1.56	2.56 ± 1.26	1.40	4.07 ± 2.71
Mitochondrial ribosomal protein L39 (*MRPL39*)	21	7.48E-04	1.44	1.25 ± 0.08	1.04	1.40 ± 0.27	1.24	2.41 ± 1.14	1.41	1.91 ± 0.29
Interferon gamma receptor 2 (*IFNGR2*)	21	8.16E-04	1.36	2.21 ± 0.29	1.36	1.78 ± 0.12	1.48	1.82 ± 0.44	1.27	1.27 ± 0.41
Human homolog of ES1 (zebrafish) protein (C21orf33)	21	1.02E-03	1.48	5.44 ± 2.14	1.71	1.85 ± 0.40	1.06	1.21 ± 0.24	1.99	1.38 ± 0.16
Chaperonin containing TCP1, subunit 8 (theta) (*CCT8*)	21	1.45E-03	1.38	2.51 ± 0.44	1.29	3.64 ± 0.92	1.94	1.09 ± 0.07	1.77	0.75 ± 0.04
Tryptophan rich basic protein (*WRB*)	21	2.18E-03	1.89	5.21 ± 2.08	1.28	1.94 ± 0.39	1.83	2.87 ± 2.23	1.94	1.78 ± 0.44
HMT1 hnRNP methyl-transferase-like 1 (*HRMT1L1*)	21	3.62E-03	1.37	1.50 ± 0.54	1.44	1.93 ± 0.36	0.96	1.46 ± 0.50	1.45	1.65 ± 0.05

Data were normalized to the HPRT housekeeping gene. P values were derived from ANOVA of microarray data. Values for microarray data are fold-regulation. For each quantitative PCR (qPCR) experiment, values were determined by measuring samples in duplicate at multiple concentrations (mean ± standard error). Each experiment was performed independently at least three times.

**Table 10:** Quantitative real-time PCR results for selected non-chromosome 21 genes in Table 2

Gene name	Chromosome	*p *value	Cerebrum		Cerebellum		Astrocyte		Heart	
			
			Microarray	qPCR	Microarray	qPCR	Microarray	qPCR	Microarray	qPCR
Myomesin 1 (skelemin) (185 kDa) (*MYOM1*)	18	8.82E-08	BBL	-	BBL	-	BBL	-	1.40	1.25 ± 0.06
Myoglobin (*MB*)	22	1.09E-07	BBL	-	BBL	-	BBL	-	1.64	1.58 ± 0.16
Calsequestrin 2 (cardiac muscle) (*CASQ2*)	1	1.56E-07	BBL	-	BBL	-	BBL	-	1.65	2.89 ± 0.58
Ras-related associated with diabetes (*RRAD*)	16	5.06E-06	BBL	-	BBL	-	BBL	-	0.49	0.82 ± 0.08
Troponin I, cardiac (*TNNI3*)	19	5.90E-06	BBL	-	BBL	-	BBL	-	1.62	1.31 ± 0.25
Insulin-like growth factor binding protein 7 (*IGFBP7*)	4	1.12E-05	1.41	1.02 ± 0.27	0.70	1.55 ± 0.33	1.74	2.12 ± 0.08	1.53	0.77 ± 0.09
Actin, alpha 1, skeletal muscle (*ACTA1*)	1	1.20E-05	BBL	-	BBL	-	BBL	-	4.17	6.27 ± 0.59
Calcineurin-binding protein calsarcin-1 (*MYOZ2*)	4	1.22E-05	BBL	-	BBL	-	BBL	-	1.49	1.35 ± 0.09
Cardiac troponin T2 (*TNNT2*)	1	2.56E-05	BBL	-	BBL	-	BBL	-	1.34	1.31 ± 0.25
A disintegrin-like and metalloprotease (reprolysin type) with thrombospondin type 1 motif, 8 (*ADAMTS8*)	11	3.21E-05	BBL	-	BBL	-	BBL	-	0.55	0.55 ± 0.17

Data were normalized to the *HPRT *housekeeping gene. *P *values were derived using the same ANOVA model used for the microarray data. Values for microarray data are fold-regulation. For each qPCR experiment, values were determined by measuring samples in duplicate at multiple concentrations (mean ± standard error). Each experiment was performed independently at least three times. BBL, below background level; qPCR, quantitative real-time PCR.
