# Supplementary material for: Primary and secondary transcriptional effects in the developing human Down syndrome brain and heart
Source: Genome Biol. 2005 Dec 16;6(13):R107. doi: 10.1186/gb-2005-6-13-r107 (PMC1414106; doi:10.1186/gb-2005-6-13-r107)
Supplement: Additional data file 1 — Lists information on 25 samples such as race, gender, and postmortem interval. [file gb-2005-6-13-r107-S1.doc]

## Additional Table 1. Information on samples used in microarray studies.

| **Sample^a^** | **Source** | **Type** | **Age (gw)** | **Gender** | **Race** | **PMI (hrs)** |
| --- | --- | --- | --- | --- | --- | --- |
| 1521 | astrocyte | control | 18 | female | African American | 1 |
| 1479 | astrocyte | control | 17 | female | African American | 1 |
| 748 | astrocyte | TS21 | 20 | male | Caucasian | 2 |
| 1478 | astrocyte | TS21 | 18 | female | Caucasian | 4 |
| 1390 | cerebrum | control | 18 | female | African American | 1 |
| 1411 | cerebrum | control | 18 | male | African American | 1 |
| 1521 | cerebrum | control | 18 | female | African American | 1 |
| 1565 | cerebrum | control | 18 | female | African American | 2 |
| 847 | cerebrum | TS21 | 18 | female | Caucasian | 1 |
| 1218 | cerebrum | TS21 | 19 | female | Caucasian | 1 |
| 1389 | cerebrum | TS21 | 18 | male | Caucasian | 1 |
| 1478 | cerebrum | TS21 | 18 | female | Caucasian | 4 |
| 1218 | heart | TS21 | 19 | female | Caucasian | 1 |
| 1478 | heart | TS21 | 18 | female | Caucasian | 4 |
| 1390 | heart | control | 18 | female | African American | 1 |
| 1411 | heart | control | 18 | male | African American | 1 |
| 1389 | cerebellum | TS21 | 18 | male | Caucasian | 1 |
| 1478 | cerebellum | TS21 | 18 | female | Caucasian | 4 |
| 1218 | cerebellum | TS21 | 19 | female | Caucasian | 1 |
| 1390 | cerebellum | control | 18 | female | African American | 1 |
| 1411 | cerebellum | control | 18 | male | African American | 1 |
| 1521 | cerebellum | control | 18 | female | African American | 1 |
| 1390 | cerebrum | control | 18 | female | African American | 1 |
| 1411 | cerebrum | control | 18 | male | African American | 1 |
| 1521 | cerebrum | control | 18 | female | African American | 1 |

**a** Samples are identifier numbers from the Brain and Tissue Bank for Developmental Disorders, University of Maryland, Baltimore.

**Abbreviations:** TS21, trisomy 21; gw, gestational weeks; PMI, postmortem interval.
