# Supplementary material for: Primary and secondary transcriptional effects in the developing human Down syndrome brain and heart
Source: Genome Biol. 2005 Dec 16;6(13):R107. doi: 10.1186/gb-2005-6-13-r107 (PMC1414106; doi:10.1186/gb-2005-6-13-r107)
Supplement: Additional data file 2 — This table describes FDR results shown for each individual chromosome. [file gb-2005-6-13-r107-S2.doc]

| Additional Table 2 | | |  |  |  |  |
| --- | --- | --- | --- | --- | --- | --- |
| Results of test for whether individual genes assigned to any chromosome were differentially expressed in TS21 relative to euploid samples. | | | | | | |
|  |  |  |  |  |  |  |
|  |  |  |  |  |  |  |
| Chromosome | # genes | # genes pass  FDR 0.05 | % genes pass  FDR 0.05 | Present in  Table 1B | Accession | Gene name |
| 1 | 2050 | 3 | 0.146341463 | yes | NM_001232 | calsequestrin 2 (cardiac muscle) |
|  |  |  |  | yes | NM_001100 | actin, alpha 1, skeletal muscle |
|  |  |  |  | yes | X79857 | troponin T2, cardiac |
| 2 | 1342 | 0 | 0 |  |  |  |
| 3 | 1082 | 1 | 0.092421442 | yes | NM_003212 | teratocarcinoma-derived growth factor 1 |
| 4 | 752 | 2 | 0.265957447 | yes | NM_001553 | insulin-like growth factor binding protein 7 |
|  |  |  |  | yes | NM_016599 | calcineurin-binding protein calsarcin-1 |
| 5 | 897 | 0 | 0 |  |  |  |
| 6 | 1167 | 0 | 0 |  |  |  |
| 7 | 962 | 0 | 0 |  |  |  |
| 8 | 705 | 2 | 0.283687943 | no | NM_001359 | 2,4-dienoyl CoA reductase 1, mitochondrial |
|  |  |  |  | no | NM_000742 | cholinergic receptor, nicotinic, alpha polypeptide 2 |
| 9 | 747 | 1 | 0.133868809 | no | NM_002989 | small inducible cytokine subfamily A (Cys-Cys), member 21 |
| 10 | 752 | 0 | 0 |  |  |  |
| 11 | 1162 | 1 | 0.08605852 | yes | NM_007037 | a disintegrin-like and metalloprotease |
| 12 | 1096 | 0 | 0 |  |  |  |
| 13 | 358 | 1 | 0.279329609 | yes | AA459867 | olfactory receptor, family 7, subfamily E, member 12 pseudogene |
| 14 | 667 | 0 | 0 |  |  |  |
| 15 | 613 | 0 | 0 |  |  |  |
| 16 | 862 | 1 | 0.116009281 | yes | NM_004165 | Ras-related associated with diabetes |
| 17 | 1188 | 1 | 0.084175084 | yes | NM_006497 | hypermethylated in cancer 1 |
| 18 | 313 | 1 | 0.319488818 | yes | NM_003803 | myomesin 1 |
| 19 | 1318 | 1 | 0.075872534 | yes | NM_000363 | troponin I, cardiac |
| 20 | 558 | 0 | 0 |  |  |  |
| 21 | 253 | 26 | 10.27667984 |  |  |  |
| 22 | 564 | 1 | 0.177304965 | yes | NM_005368 | myoglobin |
| X | 850 | 1 | 0.117647059 | yes | NM_022144 | myodulin |
