# Supplementary material for: Primary and secondary transcriptional effects in the developing human Down syndrome brain and heart
Source: Genome Biol. 2005 Dec 16;6(13):R107. doi: 10.1186/gb-2005-6-13-r107 (PMC1414106; doi:10.1186/gb-2005-6-13-r107)
Supplement: Additional data file 3 — Detailed methods for the following topics: Expression data analysis: class prediction; Error estimation using nested cross-validation; Selection of predictor genes for classification; Expression data analysis: functional group testing; and Quantitative real-time PCR. The functional group testing section includes the description of a novel algorithm for functional group analyses. [file gb-2005-6-13-r107-S3.doc]

**Additional Methods**

***Expression data analysis: class prediction*.** To further investigate whether or not changes in gene expression are mostly isolated to chromosome 21, we attempted to classify tissue samples as TS21 or euploid controls using the expression of genes assigned to chromosome 21, and in a separate simulation using non-chromosome 21 genes. We used Partek® software for these analyses.

Our classification simulations were carried out in three parts. (1) Gene selection (variable selection). Genes were selected for use in classification based on their *p*-values from the ANOVA model specified in EQ 1. Because the optimal number of predictor genes was unknown a priori, we explored gene sets of size n = 1, 3, 5, …251, and 253 genes for each classifier. In each case the *n* genes with the smallest ANOVA *p*-values were selected for use by the classifier. A maximum of 253 genes were selected because that was the total number of genes on the chip from chromosome 21. (2) Selection of an optimal classifier. Three classification methods were evaluated: K-Nearest Neighbor (KNN) [54], Nearest “Shrunken” Centroid [55], and Discriminant Analysis. For the KNN parameters, the number of neighbors (K) was 1, 3, or 5. The similarity measures evaluated included Euclidean distance, absolute value distance (“city block”), and Pearson’s correlation. The Discriminant Analysis parameters included both linear and quadratic discriminant functions. For both nearest centroid and discriminant analysis, equally likely prior probabilities of class assignment were assumed. (3) Estimating the accuracy of the classifier (see below).

There were 1548 possible combinations of number of predictor genes and classifiers, of which 1305 were used after removing overfitting classifier configurations. Specifically, discriminant analysis models are overfitted when trained using more variables than samples, so those models which included more predictor genes than samples were omitted from the discriminant analysis simulations. In order to fairly compare the ability to classify using chromosome 21 genes versus non-chromosome 21 genes, the same model space was used for both simulations.

*Error estimation using nested cross-validation.* In order to find the optimal number of genes, classifier, and classifier parameters while producing accuracy estimates for prediction, a two-level nested cross-validation was performed as follows. An “outer” ten-fold cross-validation was used to obtain classification accuracy estimates, while a nested, “inner”, nine-fold cross-validation was used to select genes and tune classifier parameters. Additionally, since the 25 tissue samples come from only ten subjects, we used a ten-fold outer “leave-one-subject-out” outer cross-validation. At each step of the outer cross-validation, with all samples of a single subject held out as test cases, the samples from the remaining nine subjects were used to determine the optimal predictor genes, classifier type, and other classifier parameters to be applied to the held out test samples. To do so, a nine-fold leave-one-subject-out inner cross-validation was applied to each of the 1305 classifier configurations. The classifier that performed the best in the inner cross-validation was applied to the samples for the subject held-out in the outer cross-validation. In the case of ties for the best classifier from the inner cross-validation, all of the tied classifiers were applied to the samples held out in the outer cross-validation and an average classification rate of the tied models was used for the error estimate for that test subject. The classification rate for each subject was additionally weighted by the number of samples from that subject. Thus an “inner” nine-fold cross-validation was performed in order to select predictor genes and optimal classifier parameters, and an “outer” ten-fold cross-validation was used to produce overall accuracy estimates for the classifier. We refer to this as a “10×9 nested cross-validation”. The nested cross-validation procedure described above ensures that the test samples used for final accuracy estimation were not used to select the predictor genes, the number of predictor genes, the model type, or the model parameters.

*Selection of predictor genes for classification.* Gene selection was performed by using the same ANOVA model as in EQ 1. The genes with the smallest *p*-values for a difference between TS21 and euploid controls were selected. Because the tissues studied have substantially different expression profiles, we included the tissue type as a predictor in addition to the expression values themselves. The tissue type was “dummy-encoded” and included as predictors in the model as follows: The tissue type was encoded using four binary variables, one variable for each of the four tissue types. Each variable was set to zero except for the one corresponding to the tissue for that sample. For that variable, a value of 250 was used as a positive indicator of that tissue type (the value of 250 was chosen to give the dummy variables approximately the same variance as the average gene). These four variables that encode the tissue type were included in every classifier in addition to the selected genes.

The classification accuracy when using non-chromosome 21 genes was approximately equal to the accuracy expected by chance (Table 4). An accuracy estimate of less than 50% can occur, because certain classifiers have a tendency to produce error estimates worse than 50% using cross-validation when discriminatory power is low and the sample size is small. Consider the behavior of a one nearest neighbor (1NN) classifier for example a data set with only two TS21 samples and two controls. If one sample is held out as a test case in cross-validation, 2/3 of the remaining samples are from the other class. Thus the 1NN classifier will have a 2/3 probability of assigning the test case to the wrong class. The expected accuracy estimate by chance alone for this small sample size is 33.33%. In summary, our ability to classify tissue samples using chromosome 21 genes was nearly perfect, and our ability to classify the tissue samples using non-chromosome 21 genes was approximately equal to random chance.

***Expression data analysis: functional group testing.*** Most of the probe sets on the Affymetrix GeneChip® human U133A microarray can be assigned to one or more functional groups with a unique ID number based upon Gene Ontology (GO) annotations [31, 32](www.geneontology.org). GO IDs are organized in a tree-like structure via parent-child relationships. The top level has only one group: "Gene_Ontology", which is then sub-divided into three groups at the second level, including biological_process, cellular_component, and molecular_function. To assess the statistical significance of gene expression differences in distinct functional groups, we implemented a novel t-test procedure that we named a 5T analysis (tree-travel, transform, t-test). This algorithm differs from web-based tools such as GoMiner [56], FatiGO [57], GO:TermFinder [58], or GOTree Machine [59], which define genes as either regulated or not, and employ a Fisher’s exact test or hypergeometric distribution analysis. Under the usual assumptions, namely independence and normality of the error, a t-test offers more power than a test with a dichotomized outcome. Our algorithm also differs from methods such as MAPPFinder [60] that assess the significance of a user-defined, predetermined set of genes of interest.

The first step is the tree-travel step. For each probe set, we parsed its GO annotations, and generated a list of functional groups located in- the top six levels of GO tree structure. For example, if a probe set was assigned to a functional group at the ninth level of the tree structure, it was then traced back to the functional group at the sixth level of the tree and assigned to that functional group in the final list.

In the transform step, we then focused on the functional groups located at the sixth level and above in the GO tree. For each functional group, a list of probe sets assigned to this functional group and a list of probe sets not assigned to this functional group (“non-group members”) are thus generated. To avoid bias due to genes with signals below background levels, probe sets with raw signal intensities ≤50 were excluded from the following analysis.

In the t-test step, for each functional group with three or more members in a tissue/cell type, we had two lists of probe sets: one list consisted of the log ratio values (log base = 10) of all the probe sets assigned to this functional group, and the other list consisted of log ratio values from all other probe sets. We performed a t-test on these two groups of log ratio values. The process was repeated for all the functional groups. All the functional groups in a tissue/cell type were then sorted based on their *p*-values from the t-tests. As a conservative correction for multiple comparisons we set the significance level at 0.05 divided by the number of functional groups (see Tables 5-8). To avoid discarding potentially useful information, we also performed Wilcoxon’s rank test to assess the statistical significance of differentially regulated functional groups having only one or two members.

We also applied an alternative statistical test to the data based upon a permutation principle. We started with a list of probe sets assigned to a particular functional group. We then randomly selected an equal number of probe sets from all probe sets on the microarray and calculated the mean log ratio values. This random selection was repeated for 100 times. The average of the mean log ratio values was calculated, and compared to the mean log ratio value of that particular functional group. The permutation test was performed on all functional groups.

The 5T data analysis method was implemented in Perl on a Dual-Processor RedHat Fedora Linux server using a MySQL version of the GO database.

***Quantitative real-time PCR.*** Total RNA was isolated from frozen tissues or astrocytes using RNeasy® Midi Kit (Qiagen) and followed by cDNA synthesis using Invitrogen SuperScript™ First-Strand System for RT-PCR (Invitrogen Life Technologies). Quantitative real-time PCR was performed by a 7900HT Sequence Detector System (Applied Biosystems) or LightCycler (Roche Molecular Biochemicals). Specific primers for the target genes were designed by Primer Express software (Applied Biosystems) or Primer3 program (http://frodo.wi.mit.edu). Where possible, the primers were designed to span an intron to ensure that there was no genomic DNA contamination in the cDNA samples. Primer sequences are listed in Additional data file 8. Real-time PCR was carried out using SYBR® Green PCR Core Reagents (Applied Biosystems) for the 7900HT or LightCycler FastStart DNA Master SYBR Green (Roche) for the LightCycler in a final volume of 20μl. The PCR conditions for 7900HT were 50°C for 2 min, 95°C for 10 min, and 40-50 cycles of 95°C for 15 sec, 60°C for 30 sec, and 72°C for 30 sec. Conditions for the LightCycler were 95°C for 10 min and 40-50 cycles of 95°C for 15 sec, 60°C for 10 sec, and 72°C for 15 sec. The expression level of hypoxanthine phosphoribosyltransferase (*HPRT*) housekeeping gene was used for normalization. For the 7900HT, real-time PCR for *HPRT* was performed using a pre-mixture of fluorescent probe and primers and TaqMan® Universal PCR Master Mix (Applied Biosystems). Probes contained VIC at the 5’ end as a reporter and TAMRA at 3’ end as a quencher. The conditions were 50°C for 2 min, 95°C for 10 min, and 45 cycles of 95°C for 15 sec and 60°C for 1 min. For the LightCycler, the LightCycler-h-*HPRT* Housekeeping Gene Set and LightCycler DNA Master Hybridization Probes (Roche) were used for the amplification of *HPRT*. The hybridization probes consisted of two oligonucleotides specific for internal sequence of amplified product. One probe was labeled with LightCycler Red 640 at the 5’ end and the other one was labeled with fluorescein at the 3’ end. The condition was 95°C for 10 min and 45 cycles of 95°C for 10 sec, 55°C for 15 sec, and 72°C for 10 sec.

We analyzed the data with SDS 2.1 (Applied Biosystems) or LightCycler software. Briefly, three serial ten-fold dilutions of cDNA were amplified in duplicates to construct standard curves. Standard curves generated by the software were used for extrapolation of expression level for the unknown samples based on their threshold cycle (Ct) values. All amplifications of unknown samples were in the linear range. For each reaction, melting curves and agarose gel electrophoresis of PCR products were used to verify the identity of the amplification products. All experiments were performed with at least three independent PCR reactions.
