# Supplementary material for: Primary and secondary transcriptional effects in the developing human Down syndrome brain and heart
Source: Genome Biol. 2005 Dec 16;6(13):R107. doi: 10.1186/gb-2005-6-13-r107 (PMC1414106; doi:10.1186/gb-2005-6-13-r107)
Supplement: Additional data file 4 — Figure legends for the Additional data file 5 and 7 figures. [file gb-2005-6-13-r107-S4.doc]

## Additional Figure Legends

**Additional** **Figure 1.** Permutation test on GO functional groups. For each functional group, a two sample t-test was carried out, testing for a difference in expression for genes associated with this functional group, compared to all other observed gene expression levels. Results are shown for the distribution of p-values (y-axis) for all functional groups in astrocyte, cerebellum, cerebrum, and heart (x-axis). Data from 100 random shuffles of the observed gene expression values are indicated (100 black lines); the original data are indicated (red line in each panel).

**Additional** **Figure 2.** Relative amounts of *ZNF294* transcripts present in the fetal TS21 and euploid cerebrum samples detected by quantitative real-time PCR. Expression levels were normalized to the *HPRT* housekeeping gene. Error bars represent standard error of the mean.
