# Supplementary material for: Primary and secondary transcriptional effects in the developing human Down syndrome brain and heart
Source: Genome Biol. 2005 Dec 16;6(13):R107. doi: 10.1186/gb-2005-6-13-r107 (PMC1414106; doi:10.1186/gb-2005-6-13-r107)
Supplement: Additional data file 6 — This table provides results of a Wilcoxon rank test which is appropriate for functional groups having a small size. [file gb-2005-6-13-r107-S6.doc]

Additional Table 3: Results of Wilcoxon rank test for analysis of functional group regulation.

Abbreviation: fg, functional group; GO, Gene Ontology; U, underexpressed; O, overexpressed

Explanation of headers: rank refers to the place among the approximately 10,000 probesets analyzed (9,583 in astrocytes; 10,497 in cerebellum; 9,704 in cerebrum; 10,624 in heart).

For example, a rank of 19 means there were 18 gene expression measurements smaller than the one observed

Direction refers to direction on regulation (underexpressed, U; overexpressed, O)

astrocyte

|  | Source | # genes per fg | GO group | p value | rank | direction |  |
| --- | --- | --- | --- | --- | --- | --- | --- |
| 1 | astrocyte | 1 | GO:0030295 | 0.004 | 19 | U |  |
| 2 | astrocyte | 1 | GO:0015232 | 0.0083 | 40 | U |  |
| 3 | astrocyte | 1 | GO:0007321 | 0.0163 | 78 | U |  |
| 4 | astrocyte | 1 | GO:0017128 | 0.0213 | 102 | O |  |
| 5 | astrocyte | 1 | GO:0005189 | 0.0223 | 107 | O |  |
| 6 | astrocyte | 1 | GO:0048154 | 0.024 | 115 | O |  |
| 7 | astrocyte | 1 | GO:0048155 | 0.024 | 115 | O |  |
| 8 | astrocyte | 1 | GO:0048156 | 0.024 | 115 | O |  |
| 9 | astrocyte | 1 | GO:0001515 | 0.0248 | 119 | U |  |
| 10 | astrocyte | 1 | GO:0007499 | 0.0261 | 125 | O |  |
| 11 | astrocyte | 1 | GO:0007638 | 0.0311 | 149 | U |  |
| 12 | astrocyte | 1 | GO:0001772 | 0.0313 | 150 | U |  |
| 13 | astrocyte | 1 | GO:0042608 | 0.0313 | 150 | U |  |
| 14 | astrocyte | 1 | GO:0019208 | 0.0342 | 164 | O |  |
| 15 | astrocyte | 1 | GO:0046332 | 0.0342 | 164 | O |  |
| 16 | astrocyte | 1 | GO:0004978 | 0.0376 | 180 | U |  |
| 17 | astrocyte | 1 | GO:0008281 | 0.0392 | 188 | U |  |
| 18 | astrocyte | 1 | GO:0016923 | 0.0493 | 236 | O |  |
| 19 | astrocyte | 1 | GO:0008395 | 0.0526 | 252 | U |  |
| 20 | astrocyte | 1 | GO:0015485 | 0.0532 | 255 | U |  |
|  | | | |  | | | |
|  | Source | # genes per fg | GO group | p value | rank1 | rank2 | direction |
| 1 | astrocyte | 2 | GO:0008147 | 0.0001 | 37 | 51 | O |
| 2 | astrocyte | 2 | GO:0007589 | 0.0004 | 32 | 170 | U |
| 3 | astrocyte | 2 | GO:0008048 | 0.0004 | 32 | 170 | U |
| 4 | astrocyte | 2 | GO:0017147 | 0.0073 | 34 | 788 | O |
| 5 | astrocyte | 2 | GO:0030935 | 0.0084 | 234 | 644 | O |
| 6 | astrocyte | 2 | GO:0004666 | 0.0142 | 377 | 766 | U |
| 7 | astrocyte | 2 | GO:0007494 | 0.0156 | 227 | 972 | U |
| 8 | astrocyte | 2 | GO:0030377 | 0.0188 | 159 | 1155 | O |
| 9 | astrocyte | 2 | GO:0005698 | 0.0191 | 600 | 724 | U |
| 10 | astrocyte | 2 | GO:0030695 | 0.0203 | 619 | 746 | O |
| 11 | astrocyte | 2 | GO:0050664 | 0.0424 | 167 | 1806 | U |
| 12 | astrocyte | 2 | GO:0015035 | 0.0516 | 614 | 1564 | O |
| 13 | astrocyte | 2 | GO:0045595 | 0.0633 | 951 | 1460 | U |
| 14 | astrocyte | 2 | GO:0005811 | 0.0767 | 107 | 2547 | O |
| 15 | astrocyte | 2 | GO:0007595 | 0.0818 | 173 | 2569 | O |
| 16 | astrocyte | 2 | GO:0005154 | 0.0883 | 1412 | 1437 | O |
| 17 | astrocyte | 2 | GO:0009320 | 0.103 | 1173 | 1903 | U |
| 18 | astrocyte | 2 | GO:0016899 | 0.1147 | 1454 | 1792 | U |
| 19 | astrocyte | 2 | GO:0005971 | 0.1307 | 1365 | 2100 | U |
| 20 | astrocyte | 2 | GO:0004325 | 0.1375 | 1665 | 1889 | O |
|  | | | |  | | | |
|  | Source | # genes per fg | GO group | -log10 p value | rank1 | rank2 |  |
| 1 | Astrocyte | 141 | GO:0004930 | 8.82 | 3395 | 4813 |  |
| 2 | Astrocyte | 15 | GO:0005581 | 8.45 | 9006 | 4785 |  |
| 3 | Astrocyte | 49 | GO:0005201 | 7.85 | 7028 | 4781 |  |
| 4 | Astrocyte | 14 | GO:0005583 | 7.59 | 8907 | 4786 |  |
| 5 | Astrocyte | 307 | GO:0005783 | 6.44 | 5582 | 4766 |  |
| 6 | Astrocyte | 734 | GO:0005887 | 6.02 | 4311 | 4832 |  |
| 7 | Astrocyte | 381 | GO:0006952 | 5.16 | 4168 | 4818 |  |
| 8 | Astrocyte | 120 | GO:0005578 | 5.14 | 5918 | 4778 |  |
| 9 | Astrocyte | 66 | GO:0007276 | 4.72 | 3342 | 4802 |  |
| 10 | Astrocyte | 73 | GO:0005216 | 4.67 | 3421 | 4803 |  |
| 11 | Astrocyte | 482 | GO:0007166 | 4.51 | 4280 | 4819 |  |
| 12 | Astrocyte | 122 | GO:0043232 | 4.14 | 3804 | 4805 |  |
| 13 | Astrocyte | 23 | GO:0005604 | 4.06 | 7052 | 4787 |  |
| 14 | Astrocyte | 82 | GO:0007517 | 3.7 | 5923 | 4782 |  |
| 15 | Astrocyte | 16 | GO:0008643 | 3.66 | 7345 | 4788 |  |
| 16 | Astrocyte | 229 | GO:0006811 | 3.6 | 4130 | 4808 |  |
| 17 | Astrocyte | 8 | GO:0019956 | 3.56 | 1237 | 4795 |  |
| 18 | Astrocyte | 5 | GO:0001527 | 3.46 | 9218 | 4790 |  |
| 19 | Astrocyte | 10 | GO:0016917 | 3.43 | 1678 | 4795 |  |
| 20 | Astrocyte | 111 | GO:0019226 | 3.43 | 3863 | 4803 |  |
|  | | | |  | | | |
|  | Source | # genes per fg | GO group | -log10 p value | mean1 | mean2 |  |
| 1 | astrocyte | 15 | GO:0005581 | 6.11 | 0.33 | -0.01 |  |
| 2 | astrocyte | 307 | GO:0005783 | 6.05 | 0.03 | -0.02 |  |
| 3 | astrocyte | 14 | GO:0005583 | 5.3 | 0.29 | -0.01 |  |
| 4 | astrocyte | 122 | GO:0043232 | 5.1 | -0.06 | -0.01 |  |
| 5 | astrocyte | 141 | GO:0004930 | 4.9 | -0.09 | -0.01 |  |
| 6 | astrocyte | 66 | GO:0007276 | 4.61 | -0.08 | -0.01 |  |
| 7 | astrocyte | 734 | GO:0005887 | 4.59 | -0.04 | -0.01 |  |
| 8 | astrocyte | 11 | GO:0016862 | 3.66 | 0.04 | -0.01 |  |
| 9 | astrocyte | 16 | GO:0008643 | 3.62 | 0.1 | -0.01 |  |
| 10 | astrocyte | 309 | GO:0006259 | 3.4 | -0.04 | -0.01 |  |
| 11 | astrocyte | 120 | GO:0005578 | 3.17 | 0.09 | -0.02 |  |
| 12 | astrocyte | 82 | GO:0007517 | 3.17 | 0.06 | -0.01 |  |
| 13 | astrocyte | 381 | GO:0006952 | 3.07 | -0.04 | -0.01 |  |
| 14 | astrocyte | 227 | GO:0007155 | 3.05 | 0.04 | -0.01 |  |
| 15 | astrocyte | 12 | GO:0045012 | 3.04 | -0.12 | -0.01 |  |
| 16 | astrocyte | 8 | GO:0019956 | 2.94 | -0.21 | -0.01 |  |
| 17 | astrocyte | 49 | GO:0005201 | 2.94 | 0.19 | -0.01 |  |
| 18 | astrocyte | 10 | GO:0016917 | 2.9 | -0.17 | -0.01 |  |
| 19 | astrocyte | 7 | GO:0005815 | 2.86 | -0.13 | -0.01 |  |
| 20 | astrocyte | 6 | GO:0008538 | 2.85 | -0.11 | -0.01 |  |

cerebellum

|  | Source | # genes per fg | GO group | p value | rank | direction |  |
| --- | --- | --- | --- | --- | --- | --- | --- |
| 1 | cerebellum | 1 | GO:0016019 | 0.0051 | 27 | U |  |
| 2 | cerebellum | 1 | GO:0016910 | 0.0076 | 40 | O |  |
| 3 | cerebellum | 1 | GO:0045445 | 0.0076 | 40 | O |  |
| 4 | cerebellum | 1 | GO:0009591 | 0.0084 | 44 | O |  |
| 5 | cerebellum | 1 | GO:0030936 | 0.0269 | 141 | U |  |
| 6 | cerebellum | 1 | GO:0005012 | 0.0299 | 157 | O |  |
| 7 | cerebellum | 1 | GO:00049781 | 0.0326 | 171 | U |  |
| 8 | cerebellum | 1 | GO:00152321 | 0.0343 | 180 | U |  |
| 9 | cerebellum | 1 | GO:0005972 | 0.0488 | 256 | O |  |
| 10 | cerebellum | 1 | GO:0008001 | 0.0488 | 256 | O |  |
| 11 | cerebellum | 1 | GO:0005335 | 0.0549 | 288 | O |  |
| 12 | cerebellum | 1 | GO:0015222 | 0.0549 | 288 | O |  |
| 13 | cerebellum | 1 | GO:0030567 | 0.0549 | 288 | U |  |
| 14 | cerebellum | 1 | GO:0017015 | 0.0614 | 322 | U |  |
| 15 | cerebellum | 1 | GO:0008514 | 0.0646 | 339 | U |  |
| 16 | cerebellum | 1 | GO:00481541 | 0.0655 | 344 | O |  |
| 17 | cerebellum | 1 | GO:00481551 | 0.0655 | 344 | O |  |
| 18 | cerebellum | 1 | GO:00481561 | 0.0655 | 344 | O |  |
| 19 | cerebellum | 1 | GO:0005582 | 0.0716 | 376 | U |  |
| 20 | cerebellum | 1 | GO:0008046 | 0.0726 | 381 | U |  |
|  | | | |  | | | |
|  | Source | # genes per fg | GO group | p value | rank1 | rank2 | direction |
| 1 | cerebellum | 2 | GO:0008367 | 0.0004 | 27 | 176 | O |
| 2 | cerebellum | 2 | GO:0005066 | 0.0029 | 214 | 355 | U |
| 3 | cerebellum | 2 | GO:0005618 | 0.0062 | 320 | 507 | O |
| 4 | cerebellum | 2 | GO:0005173 | 0.0229 | 48 | 1542 | U |
| 5 | cerebellum | 2 | GO:0007515 | 0.0265 | 425 | 1284 | O |
| 6 | cerebellum | 2 | GO:0008389 | 0.0453 | 896 | 1340 | O |
| 7 | cerebellum | 2 | GO:0005930 | 0.0487 | 1036 | 1282 | O |
| 8 | cerebellum | 2 | GO:0004126 | 0.0505 | 129 | 2231 | O |
| 9 | cerebellum | 2 | GO:0046875 | 0.052 | 967 | 1427 | U |
| 10 | cerebellum | 2 | GO:0004161 | 0.0526 | 695 | 1713 | O |
| 11 | cerebellum | 2 | GO:0016363 | 0.0529 | 822 | 1593 | U |
| 12 | cerebellum | 2 | GO:0005903 | 0.0546 | 1087 | 1366 | O |
| 13 | cerebellum | 2 | GO:00075951 | 0.0628 | 1079 | 1553 | O |
| 14 | cerebellum | 2 | GO:0015216 | 0.0701 | 557 | 2223 | O |
| 15 | cerebellum | 2 | GO:0008393 | 0.0837 | 514 | 2524 | O |
| 16 | cerebellum | 2 | GO:0016810 | 0.0864 | 631 | 2455 | O |
| 17 | cerebellum | 2 | GO:0009053 | 0.0928 | 1402 | 1796 | O |
| 18 | cerebellum | 2 | GO:0001772 | 0.0959 | 1288 | 1963 | O |
| 19 | cerebellum | 2 | GO:0005929 | 0.0996 | 610 | 2703 | O |
| 20 | cerebellum | 2 | GO:0007632 | 0.1144 | 1074 | 2477 | O |
|  | | | |  | | | |
|  | Source | # genes per fg | GO group | -log10 p value | rank1 | rank2 |  |
| 1 | cerebellum | 933 | GO:0005887 | 1.43E+01 | 5991 | 5177 |  |
| 2 | cerebellum | 469 | GO:0003723 | 1.26E+01 | 4248 | 5296 |  |
| 3 | cerebellum | 170 | GO:0019226 | 1.01E+01 | 6746 | 5224 |  |
| 4 | cerebellum | 212 | GO:0004930 | 9.90E+00 | 6574 | 5222 |  |
| 5 | cerebellum | 430 | GO:0006952 | 9.76E+00 | 6162 | 5210 |  |
| 6 | cerebellum | 2194 | GO:0005634 | 9.10E+00 | 4895 | 5342 |  |
| 7 | cerebellum | 592 | GO:0007166 | 7.57E+00 | 5922 | 5209 |  |
| 8 | cerebellum | 212 | GO:0003735 | 7.09E+00 | 4144 | 5272 |  |
| 9 | cerebellum | 1126 | GO:0003677 | 6.90E+00 | 4798 | 5303 |  |
| 10 | cerebellum | 286 | GO:0016070 | 6.41E+00 | 4352 | 5274 |  |
| 11 | cerebellum | 337 | GO:0007399 | 5.89E+00 | 6035 | 5223 |  |
| 12 | cerebellum | 495 | GO:0003676 | 5.84E+00 | 4608 | 5281 |  |
| 13 | cerebellum | 147 | GO:0005840 | 5.79E+00 | 4059 | 5266 |  |
| 14 | cerebellum | 120 | GO:0005216 | 5.67E+00 | 6553 | 5234 |  |
| 15 | cerebellum | 1596 | GO:0006139 | 5.53E+00 | 4922 | 5308 |  |
| 16 | cerebellum | 84 | GO:0000785 | 4.76E+00 | 3835 | 5260 |  |
| 17 | cerebellum | 324 | GO:0006996 | 4.32E+00 | 4575 | 5270 |  |
| 18 | cerebellum | 13 | GO:0005583 | 4.23E+00 | 1875 | 5253 |  |
| 19 | cerebellum | 15 | GO:0016917 | 4.17E+00 | 8364 | 5245 |  |
| 20 | cerebellum | 886 | GO:0044260 | 4.02E+00 | 4869 | 5284 |  |
|  | | | |  | | | |
|  | Source | # genes per fg | GO group | -log10 p value | mean1 | mean2 |  |
| 1 | cerebellum | 933 | GO:0005887 | 1.09E+01 | 0.03 | 0 |  |
| 2 | cerebellum | 469 | GO:0003723 | 1.02E+01 | -0.02 | 0.01 |  |
| 3 | cerebellum | 212 | GO:0003735 | 7.95E+00 | -0.03 | 0.01 |  |
| 4 | cerebellum | 212 | GO:0004930 | 7.89E+00 | 0.06 | 0 |  |
| 5 | cerebellum | 170 | GO:0019226 | 7.48E+00 | 0.06 | 0 |  |
| 6 | cerebellum | 2194 | GO:0005634 | 7.45E+00 | -0.01 | 0.01 |  |
| 7 | cerebellum | 592 | GO:0007166 | 6.65E+00 | 0.03 | 0 |  |
| 8 | cerebellum | 147 | GO:0005840 | 6.33E+00 | -0.03 | 0.01 |  |
| 9 | cerebellum | 430 | GO:0006952 | 5.97E+00 | 0.04 | 0 |  |
| 10 | cerebellum | 1596 | GO:0006139 | 5.27E+00 | -0.01 | 0.01 |  |
| 11 | cerebellum | 337 | GO:0007399 | 5.02E+00 | 0.04 | 0 |  |
| 12 | cerebellum | 13 | GO:0008304 | 4.60E+00 | -0.05 | 0.01 |  |
| 13 | cerebellum | 286 | GO:0016070 | 4.34E+00 | -0.02 | 0.01 |  |
| 14 | cerebellum | 15 | GO:0016917 | 4.19E+00 | 0.1 | 0.01 |  |
| 15 | cerebellum | 1126 | GO:0003677 | 4.14E+00 | -0.01 | 0.01 |  |
| 16 | cerebellum | 84 | GO:0000785 | 4.09E+00 | -0.04 | 0.01 |  |
| 17 | cerebellum | 495 | GO:0003676 | 3.97E+00 | -0.01 | 0.01 |  |
| 18 | cerebellum | 12 | GO:0000228 | 3.71E+00 | -0.06 | 0.01 |  |
| 19 | cerebellum | 323 | GO:0005783 | 3.63E+00 | -0.02 | 0.01 |  |
| 20 | cerebellum | 126 | GO:0043232 | 3.54E+00 | -0.03 | 0.01 |  |

cerebrum

|  | Source | # genes per fg | GO group | p value | rank | direction |  |
| --- | --- | --- | --- | --- | --- | --- | --- |
| 1 | cerebrum | 1 | GO:0005930 | 0.0078 | 38 | U |  |
| 2 | cerebrum | 1 | GO:0005330 | 0.0124 | 60 | O |  |
| 3 | cerebrum | 1 | GO:00051891 | 0.0183 | 89 | O |  |
| 4 | cerebrum | 1 | GO:0004657 | 0.021 | 102 | O |  |
| 5 | cerebrum | 1 | GO:0005157 | 0.0221 | 107 | U |  |
| 6 | cerebrum | 1 | GO:0004055 | 0.0239 | 116 | O |  |
| 7 | cerebrum | 1 | GO:0005555 | 0.0254 | 123 | U |  |
| 8 | cerebrum | 1 | GO:0005577 | 0.0272 | 132 | U |  |
| 9 | cerebrum | 1 | GO:0008048 | 0.0289 | 140 | U |  |
| 10 | cerebrum | 1 | GO:00049782 | 0.0311 | 151 | O |  |
| 11 | cerebrum | 1 | GO:0008437 | 0.0342 | 166 | O |  |
| 12 | cerebrum | 1 | GO:00053351 | 0.0385 | 187 | U |  |
| 13 | cerebrum | 1 | GO:00152221 | 0.0385 | 187 | U |  |
| 14 | cerebrum | 1 | GO:0005142 | 0.0592 | 287 | U |  |
| 15 | cerebrum | 1 | GO:0030219 | 0.0592 | 287 | U |  |
| 16 | cerebrum | 1 | GO:0004203 | 0.0608 | 295 | U |  |
| 17 | cerebrum | 1 | GO:00154851 | 0.067 | 325 | U |  |
| 18 | cerebrum | 1 | GO:0017127 | 0.067 | 325 | U |  |
| 19 | cerebrum | 1 | GO:0030288 | 0.0678 | 329 | O |  |
| 20 | cerebrum | 1 | GO:0008225 | 0.0686 | 333 | U |  |
|  | | | |  | | | |
|  | Source | # genes per fg | GO group | p value | rank1 | rank2 | direction |
| 1 | cerebrum | 2 | GO:0015067 | 0.0006 | 80 | 157 | O |
| 2 | cerebrum | 2 | GO:00056181 | 0.0047 | 297 | 369 | O |
| 3 | cerebrum | 2 | GO:0001515 | 0.005 | 182 | 505 | O |
| 4 | cerebrum | 2 | GO:00455951 | 0.0122 | 47 | 1024 | O |
| 5 | cerebrum | 2 | GO:0006854 | 0.0165 | 474 | 775 | U |
| 6 | cerebrum | 2 | GO:00093201 | 0.0252 | 529 | 1012 | O |
| 7 | cerebrum | 2 | GO:0003762 | 0.026 | 291 | 1275 | U |
| 8 | cerebrum | 2 | GO:0001850 | 0.0277 | 370 | 1246 | U |
| 9 | cerebrum | 2 | GO:0004875 | 0.0277 | 370 | 1246 | U |
| 10 | cerebrum | 2 | GO:0008509 | 0.0316 | 450 | 1277 | U |
| 11 | cerebrum | 2 | GO:0040014 | 0.0501 | 786 | 1387 | U |
| 12 | cerebrum | 2 | GO:00506641 | 0.0508 | 23 | 2165 | U |
| 13 | cerebrum | 2 | GO:0007444 | 0.055 | 146 | 2130 | U |
| 14 | cerebrum | 2 | GO:00056981 | 0.0618 | 471 | 1943 | U |
| 15 | cerebrum | 2 | GO:0004096 | 0.0639 | 827 | 1627 | O |
| 16 | cerebrum | 2 | GO:0017151 | 0.0803 | 346 | 2404 | U |
| 17 | cerebrum | 2 | GO:0051180 | 0.1351 | 1129 | 2438 | U |
| 18 | cerebrum | 2 | GO:0017069 | 0.1362 | 858 | 2724 | U |
| 19 | cerebrum | 2 | GO:0005517 | 0.1386 | 1735 | 1879 | U |
| 20 | cerebrum | 2 | GO:00059711 | 0.1394 | 1577 | 2047 | O |
|  | | | |  | | | |
|  | Source | # genes per fg | GO group | -log10 p value | rank1 | rank2 |  |
| 1 | cerebrum | 417 | GO:0005739 | Inf | 6013 | 4800 |  |
| 2 | cerebrum | 86 | GO:0015077 | 9.12 | 6703 | 4836 |  |
| 3 | cerebrum | 1495 | GO:0006139 | 8.97 | 4446 | 4927 |  |
| 4 | cerebrum | 299 | GO:0016491 | 8.32 | 5786 | 4823 |  |
| 5 | cerebrum | 2072 | GO:0005634 | 6.93 | 4563 | 4931 |  |
| 6 | cerebrum | 558 | GO:0003700 | 5.75 | 4302 | 4886 |  |
| 7 | cerebrum | 485 | GO:0003676 | 5.66 | 4265 | 4883 |  |
| 8 | cerebrum | 31 | GO:0003954 | 5.2 | 7121 | 4845 |  |
| 9 | cerebrum | 49 | GO:0005102 | 4.88 | 6592 | 4844 |  |
| 10 | cerebrum | 1071 | GO:0003677 | 4.68 | 4509 | 4895 |  |
| 11 | cerebrum | 166 | GO:0004930 | 4.54 | 3951 | 4868 |  |
| 12 | cerebrum | 25 | GO:0004129 | 3.74 | 6946 | 4847 |  |
| 13 | cerebrum | 167 | GO:0005576 | 3.62 | 4063 | 4866 |  |
| 14 | cerebrum | 147 | GO:0009308 | 3.27 | 5646 | 4840 |  |
| 15 | cerebrum | 15 | GO:0030106 | 3.16 | 7304 | 4849 |  |
| 16 | cerebrum | 466 | GO:0003723 | 3.06 | 4431 | 4874 |  |
| 17 | cerebrum | 4 | GO:0004667 | 3.02 | 227 | 4854 |  |
| 18 | cerebrum | 304 | GO:0006091 | 3.01 | 5374 | 4836 |  |
| 19 | cerebrum | 73 | GO:0008083 | 3 | 3778 | 4861 |  |
| 20 | cerebrum | 35 | GO:0046872 | 2.93 | 6387 | 4847 |  |
|  | | | |  | | | |
|  | Source | # genes per fg | GO group | -log10 p value | mean1 | mean2 |  |
| 1 | cerebrum | 417 | GO:0005739 | 13.85 | 0.03 | 0 |  |
| 2 | cerebrum | 86 | GO:0015077 | 8.43 | 0.05 | 0 |  |
| 3 | cerebrum | 1495 | GO:0006139 | 6.7 | -0.02 | 0 |  |
| 4 | cerebrum | 2072 | GO:0005634 | 6.26 | -0.01 | 0 |  |
| 5 | cerebrum | 485 | GO:0003676 | 5.91 | -0.02 | 0 |  |
| 6 | cerebrum | 299 | GO:0016491 | 5.48 | 0.02 | 0 |  |
| 7 | cerebrum | 31 | GO:0003954 | 5.42 | 0.06 | 0 |  |
| 8 | cerebrum | 1071 | GO:0003677 | 4.87 | -0.02 | 0 |  |
| 9 | cerebrum | 25 | GO:0004129 | 4.07 | 0.05 | 0 |  |
| 10 | cerebrum | 466 | GO:0003723 | 3.83 | -0.02 | 0 |  |
| 11 | cerebrum | 558 | GO:0003700 | 3.62 | -0.02 | 0 |  |
| 12 | cerebrum | 147 | GO:0009308 | 3.46 | 0.03 | 0 |  |
| 13 | cerebrum | 273 | GO:0016070 | 3.11 | -0.02 | 0 |  |
| 14 | cerebrum | 8 | GO:0000263 | 3.07 | 0.04 | 0 |  |
| 15 | cerebrum | 27 | GO:0004386 | 2.94 | -0.05 | 0 |  |
| 16 | cerebrum | 108 | GO:0005635 | 2.88 | -0.03 | 0 |  |
| 17 | cerebrum | 4 | GO:0008312 | 2.88 | 0.02 | 0 |  |
| 18 | cerebrum | 3 | GO:0008025 | 2.78 | 0.27 | 0 |  |
| 19 | cerebrum | 49 | GO:0005102 | 2.75 | 0.07 | 0 |  |
| 20 | cerebrum | 15 | GO:0006805 | 2.63 | -0.08 | 0 |  |

heart

|  | Source | # genes per fg | GO group | | p value | rank | direction |  |
| --- | --- | --- | --- | --- | --- | --- | --- | --- |
| 1 | heart | 1 | GO:0007320 | | 0.0006 | 3 | U |  |
| 2 | heart | 1 | GO:0006906 | | 0.0021 | 11 | U |  |
| 3 | heart | 1 | GO:0030181 | | 0.004 | 21 | O |  |
| 4 | heart | 1 | GO:0005130 | | 0.0041 | 22 | U |  |
| 5 | heart | 1 | GO:0030165 | | 0.0128 | 68 | U |  |
| 6 | heart | 1 | GO:0008301 | | 0.0194 | 103 | U |  |
| 7 | heart | 1 | GO:00154852 | | 0.0239 | 127 | O |  |
| 8 | heart | 1 | GO:00171271 | | 0.0239 | 127 | O |  |
| 9 | heart | 1 | GO:0009434 | | 0.025 | 133 | O |  |
| 10 | heart | 1 | GO:0030317 | | 0.025 | 133 | O |  |
| 11 | heart | 1 | GO:0007401 | | 0.028 | 149 | U |  |
| 12 | heart | 1 | GO:0001533 | | 0.0294 | 156 | U |  |
| 13 | heart | 1 | GO:0030216 | | 0.0294 | 156 | U |  |
| 14 | heart | 1 | GO:00082251 | | 0.0352 | 187 | O |  |
| 15 | heart | 1 | GO:0015643 | | 0.0352 | 187 | O |  |
| 16 | heart | 1 | GO:0042056 | | 0.0352 | 187 | O |  |
| 17 | heart | 1 | GO:0009315 | | 0.0369 | 196 | U |  |
| 18 | heart | 1 | GO:0004362 | | 0.0375 | 199 | O |  |
| 19 | heart | 1 | GO:0001666 | | 0.0384 | 204 | O |  |
| 20 | heart | 1 | GO:0005061 | | 0.0384 | 204 | O |  |
|  | | | | |  | | | |
|  | Source | # genes per fg | | GO group | p value | rank1 | rank2 | direction |
| 1 | heart | 2 | | GO:0015250 | 0.0025 | 89 | 445 | U |
| 2 | heart | 2 | | GO:0030492 | 0.0105 | 391 | 700 | U |
| 3 | heart | 2 | | GO:00056182 | 0.0138 | 289 | 960 | O |
| 4 | heart | 2 | | GO:0004203 | 0.0159 | 127 | 1214 | U |
| 5 | heart | 2 | | GO:00050661 | 0.0263 | 342 | 1383 | U |
| 6 | heart | 2 | | GO:0016623 | 0.0265 | 71 | 1659 | U |
| 7 | heart | 2 | | GO:0030345 | 0.0297 | 319 | 1513 | U |
| 8 | heart | 2 | | GO:0008142 | 0.0299 | 221 | 1618 | O |
| 9 | heart | 2 | | GO:0008431 | 0.033 | 929 | 1003 | U |
| 10 | heart | 2 | | GO:0004478 | 0.0398 | 876 | 1245 | O |
| 11 | heart | 2 | | GO:0015232 | 0.0474 | 222 | 2091 | O |
| 12 | heart | 2 | | GO:0030553 | 0.0573 | 55 | 2490 | U |
| 13 | heart | 2 | | GO:00163631 | 0.067 | 1163 | 1588 | O |
| 14 | heart | 2 | | GO:0009607 | 0.0703 | 931 | 1886 | U |
| 15 | heart | 2 | | GO:00075151 | 0.0776 | 1210 | 1751 | U |
| 16 | heart | 2 | | GO:00083931 | 0.0842 | 225 | 2858 | U |
| 17 | heart | 2 | | GO:0008434 | 0.0859 | 406 | 2708 | U |
| 18 | heart | 2 | | GO:00506642 | 0.087 | 665 | 2470 | O |
| 19 | heart | 2 | | GO:0007635 | 0.0873 | 1143 | 1996 | U |
| 20 | heart | 2 | | GO:0016343 | 0.0908 | 1035 | 2168 | U |
|  | | | | |  | | | |
|  | Source | # genes per fg | | GO group | -log10 p value | rank1 | rank2 |  |
| 1 | heart | 446 | | GO:0005739 | Inf | 6530 | 5259 |  |
| 2 | heart | 485 | | GO:0006952 | 11.54 | 4363 | 5358 |  |
| 3 | heart | 86 | | GO:0015077 | 8.22 | 7228 | 5297 |  |
| 4 | heart | 201 | | GO:0007267 | 7.24 | 4150 | 5335 |  |
| 5 | heart | 948 | | GO:0005887 | 6.48 | 4827 | 5360 |  |
| 6 | heart | 594 | | GO:0007166 | 5.87 | 4721 | 5348 |  |
| 7 | heart | 215 | | GO:0005576 | 5.09 | 4388 | 5332 |  |
| 8 | heart | 812 | | GO:0007165 | 4.43 | 4886 | 5348 |  |
| 9 | heart | 215 | | GO:0004930 | 4.38 | 4464 | 5330 |  |
| 10 | heart | 7 | | GO:0015101 | 3.87 | 891 | 5315 |  |
| 11 | heart | 31 | | GO:0003954 | 3.75 | 7374 | 5306 |  |
| 12 | heart | 380 | | GO:0046914 | 3.74 | 4734 | 5334 |  |
| 13 | heart | 28 | | GO:0006937 | 3.64 | 7447 | 5307 |  |
| 14 | heart | 428 | | GO:0046907 | 3.53 | 5838 | 5290 |  |
| 15 | heart | 20 | | GO:0007507 | 3.5 | 7781 | 5308 |  |
| 16 | heart | 74 | | GO:0005743 | 3.29 | 6547 | 5304 |  |
| 17 | heart | 322 | | GO:0006259 | 3.24 | 5892 | 5294 |  |
| 18 | heart | 8 | | GO:0007623 | 3.16 | 1634 | 5315 |  |
| 19 | heart | 26 | | GO:0004129 | 2.99 | 7286 | 5308 |  |
| 20 | heart | 45 | | GO:0003697 | 2.9 | 6782 | 5306 |  |
|  | | | | |  | | | |
|  | Source | # genes per fg | | GO group | -log10 p value | mean1 | mean2 |  |
| 1 | heart | 446 | | GO:0005739 | 15.18 | 0.04 | -0.01 |  |
| 2 | heart | 86 | | GO:0015077 | 9.37 | 0.07 | -0.01 |  |
| 3 | heart | 485 | | GO:0006952 | 7.64 | -0.06 | -0.01 |  |
| 4 | heart | 31 | | GO:0003954 | 5.28 | 0.07 | -0.01 |  |
| 5 | heart | 428 | | GO:0046907 | 4.63 | 0.02 | -0.01 |  |
| 6 | heart | 201 | | GO:0007267 | 3.99 | -0.07 | -0.01 |  |
| 7 | heart | 74 | | GO:0005743 | 3.78 | 0.04 | -0.01 |  |
| 8 | heart | 594 | | GO:0007166 | 3.76 | -0.04 | -0.01 |  |
| 9 | heart | 948 | | GO:0005887 | 3.71 | -0.03 | -0.01 |  |
| 10 | heart | 215 | | GO:0005576 | 3.62 | -0.06 | -0.01 |  |
| 11 | heart | 14 | | GO:0006944 | 3.33 | 0.06 | -0.01 |  |
| 12 | heart | 322 | | GO:0006259 | 3.09 | 0.02 | -0.01 |  |
| 13 | heart | 28 | | GO:0006937 | 3.03 | 0.08 | -0.01 |  |
| 14 | heart | 45 | | GO:0003697 | 2.86 | 0.06 | -0.01 |  |
| 15 | heart | 12 | | GO:0009055 | 2.85 | 0.08 | -0.01 |  |
| 16 | heart | 7 | | GO:0015101 | 2.78 | -0.27 | -0.01 |  |
| 17 | heart | 380 | | GO:0046914 | 2.73 | -0.03 | -0.01 |  |
| 18 | heart | 20 | | GO:0007507 | 2.69 | 0.11 | -0.01 |  |
| 19 | heart | 120 | | GO:0007517 | 2.6 | 0.03 | -0.01 |  |
| 20 | heart | 244 | | GO:0008565 | 2.58 | 0.02 | -0.01 |  |
