# Supplementary figures and images for: Primary and secondary transcriptional effects in the developing human Down syndrome brain and heart
Source: Genome Biol. 2005 Dec 16;6(13):R107. doi: 10.1186/gb-2005-6-13-r107 (PMC1414106; doi:10.1186/gb-2005-6-13-r107)

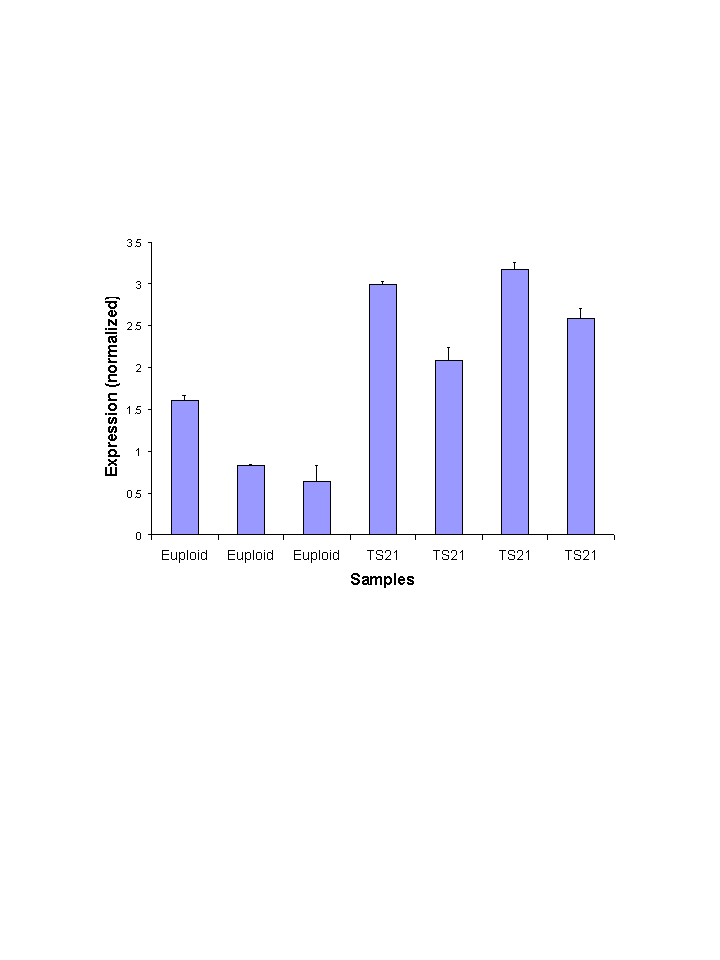

Supplement: Additional data file 7 — This figure shows a typical quantitative real-time PCR result, in which the level of a transcript is significantly up-regulated in a trisomic sample. [file gb-2005-6-13-r107-S7.tiff]
