# Supplementary material for: Primary and secondary transcriptional effects in the developing human Down syndrome brain and heart
Source: Genome Biol. 2005 Dec 16;6(13):R107. doi: 10.1186/gb-2005-6-13-r107 (PMC1414106; doi:10.1186/gb-2005-6-13-r107)
Supplement: Additional data file 8 — This table includes oligonucleotide sequences. [file gb-2005-6-13-r107-S8.doc]

## Additional Table 4. Primer sequences and other information of the quantitative real-time PCR experiments. For the other genes whose primer sequences are not listed here, real-time PCR were carried out using pre-designed TaqMan® Gene Expression Assays (Applied Biosystems). Information on these primers and assays can be found at www.appliedbiosystems.com.

| **Gene Primer Sequence Product Size System** |
| --- |
| *ATP 5O* F : 5’-GCTTGCTGAAAATGGTCGAT-3’ 336 LightCycler  R : 5’-TCACTGATGGCAGAAAACCA-3’ ABI (7900)  *APT 5J* F : 5’-CATTTGCGGAGGAACATTG-3’ 315 LightCycler  R : 5’-CAACTAATCCGTGACAAATTACCAG-3’ ABI (7900)  *PTTG1* F : 5’-TGCTGCCTAAGGAAGTTTGG-3’ 342 LightCycler  R : 5’-ACAAATGCCCAAGAGGTCAG-3’ ABI (7900)  *USP16* F : 5’-TTGAATGCTGCTCTTCATCC-3’ 307 LightCycler  R : 5’-GCTTTTTGGCATTGGTGTAA-3’ ABI (7900)  *ZNF294* F : 5’-CACGTCTCTAGTTGCTGGGCT-3’ 301 ABI (7900)  R : 5’-ACGTTCTCCAAGTTGTGAGCTG-3’  *DSCR3* F : 5’-GCCACGGAGATTCAGAACATTC-3’ 301 ABI (7900)  R : 5’-ATGCCGCTGGCTGTAGCT-3’ |
